# Corazonin signaling integrates energy homeostasis and lunar phase to regulate aspects of growth and sexual maturation in *Platynereis*

**DOI:** 10.1073/pnas.1910262116

**Published:** 2019-12-16

**Authors:** Gabriele Andreatta, Caroline Broyart, Charline Borghgraef, Karim Vadiwala, Vitaly Kozin, Alessandra Polo, Andrea Bileck, Isabel Beets, Liliane Schoofs, Christopher Gerner, Florian Raible

**Affiliations:** ^a^Max Perutz Labs, University of Vienna, A-1030 Vienna, Austria;; ^b^Animal Physiology and Neurobiology, Department of Biology, Katholieke Universiteit Leuven, 3000 Leuven, Belgium;; ^c^Department of Analytical Chemistry, University of Vienna, A-1090 Vienna, Austria

**Keywords:** corazonin, GnRH, reproduction, regeneration, lunar periodicity

## Abstract

The molecular mechanisms by which animals integrate external stimuli with internal energy balance to regulate major developmental and reproductive events still remain enigmatic. We investigated this aspect in the marine bristleworm, *Platynereis dumerilii*, a species where sexual maturation is tightly regulated by both metabolic state and lunar cycle. Our specific focus was on ligands and receptors of the gonadotropin-releasing hormone (GnRH) superfamily. Members of this superfamily are key in triggering sexual maturation in vertebrates but also regulate reproductive processes and energy homeostasis in invertebrates. Here we show that 3 of the 4 *gnrh-like* (*gnrhl*) preprohormone genes are expressed in specific and distinct neuronal clusters in the *Platynereis* brain. Moreover, ligand–receptor interaction analyses reveal a single *Platynereis* corazonin receptor (CrzR) to be activated by CRZ1/GnRHL1, CRZ2/GnRHL2, and GnRHL3 (previously classified as AKH1), whereas 2 AKH-type hormone receptors (GnRHR1/AKHR1 and GnRHR2/AKHR2) respond only to a single ligand (GnRH2/GnRHL4). *Crz1/gnrhl1* exhibits a particularly strong up-regulation in sexually mature animals, after feeding, and in specific lunar phases. Homozygous *crz1/gnrhl1* knockout animals exhibit a significant delay in maturation, reduced growth, and attenuated regeneration. Through a combination of proteomics and gene expression analysis, we identify enzymes involved in carbohydrate metabolism as transcriptional targets of CRZ1/GnRHL1 signaling. Our data suggest that *Platynereis* CRZ1/GnRHL1 coordinates glycoprotein turnover and energy homeostasis with growth and sexual maturation, integrating both metabolic and developmental demands with the worm’s monthly cycle.

## Significance

Gonadotropin Releasing Hormone (GnRH) acts as a key regulator of sexual maturation in vertebrates, and is required for the integration of environmental stimuli to orchestrate breeding cycles. Whether this integrative function is conserved across phyla remains unclear. We characterized GnRH-type signaling systems in the marine worm *Platynereis dumerilii*, in which both metabolic state and lunar cycle regulate reproduction. We find *gnrh-like* (*gnrhl*) genes upregulated in sexually mature animals, after feeding, and in specific lunar phases. Animals in which the *corazonin1/gnrhl1* gene has been disabled exhibit delays in growth, regeneration, and maturation. Molecular analyses reveal glycoprotein turnover/energy homeostasis as targets of CRZ1/GnRHL1. These findings point at an ancestral role of GnRH superfamily signaling in coordinating energy demands dictated by environmental and developmental cues.

Animals regulate development and reproduction according to environmental cues such as season, temperature, moon phase, and food availability (1–5). Despite some advances on the molecular mechanisms, the integration of multiple environmental stimuli for the regulation of reproduction and growth is still poorly understood. The marine bristleworm *Platynereis dumerilii* is well suited to tackle the endocrine basis of this integration. Worms exhibit a complex life cycle where food availability, circalunar cycle, and season regulate development and sexual maturation (6–8). Moreover, this species is molecularly accessible, and various genetic toolkits allow functional studies (9–11).

Several lines of evidence suggest that Gonadotropin-releasing hormone (GnRH)-like signaling has relevant functions in the interplay between reproduction and development, integrating both intrinsic and extrinsic factors in this balance. All bilaterians possess GnRH-like preprohormones (12, 13). This GnRH-like preprohormone superfamily is subdivided into different subfamilies, based on the phylogenetic grouping of their receptors and the historical names by which GnRH-like peptides were referred to in different animal phyla. Supported by evolutionary arguments, a recently suggested unified nomenclature (13) distinguishes 2 main receptor categories: Corazonin receptors (CrzR) and GnRH receptors. The GnRH receptors also cover the Adipokinetic hormone (AKH) and AKH-CRZ–related peptide (ACP) receptors, diversifications of a single ancestral GnRH receptor likely restricted to arthropods (13).

In vertebrates, an increase in the activity of GnRH neurons in the hypothalamus is the key event for the precise timing of sexual maturation (14). This occurs via enhanced release of 2 glycoprotein hormones, luteinizing hormone (LH) and follicle-stimulating hormone (FSH), from the anterior pituitary (15), promoting steroidogenesis and the maturation of gametes (16). Moreover, the pulsatile secretion of GnRH is crucial for the timing of the monthly reproductive cycle in women (17, 18). The GnRH system has repeatedly been suggested to play a role in the reproductive timing of semilunar/lunar synchronized spawners (19–23). However, this evidence is at present correlational. It could be linked to the spawning event per se and thus only indirectly to lunar/semilunar rhythmicity. Additional evidence for the involvement of the GnRH system in the timing of reproductive events comes primarily from studies in birds and mammals. During seasonal changes, nonphotosensitive (photorefractory or reproductively inactive) birds show a reduction in both the number and the size of GnRH immunoreactive cells (24), a condition reversed in breeding photosensitive animals (25). In mammals, the pulsatile secretion of GnRH is reduced in the nonbreeding (anestrous) season, and the reactivation of the hypothalamic–pituitary–gonadal axis is dictated by a seasonal-dependent increase in kisspeptin expression and a concomitant reduction in Gonadotropin-inhibitory hormone (GnIH) production (26–28).

Also in several invertebrate groups, preprohormones of the GnRH superfamily are involved in the regulation of different aspects of reproduction. For instance, CRZ signaling coordinates aspects of copulation and fecundity in *Drosophila* (29, 30) and steroid synthesis in *Octopus* gonads (31). Interestingly, the *Octopus* peptide was also capable to induce LH release in cultured quail pituitary cells (32). On the other hand, GnRH-like systems regulate fecundity in *Caenorhabditis elegans* (33), gonadal growth in mollusks (34), and gamete release (spawning) in *Ciona* (35). In addition, GnRH-like peptides affect body mass and feeding in *Aplysia* (36, 37), while in *Drosophila*, both CRZ and AKH regulate carbohydrate and lipid metabolism (38–41), as well as feeding (41, 42) and stress response (43, 44). Thus, regardless of their specific subtype association, invertebrate GnRH systems appear to share functions not only in reproductive control but also in other aspects of energy homeostasis.

In turn, strong interconnections between the GnRH system and metabolism can also be found in several vertebrates. *Gnrh* genes are down-regulated in animals exposed to fasting or food restriction (45, 46). Goldfish and zebrafish GnRH2 exhibits an anorexigenic function upon injection (39, 47, 48). In mammals, GnRH release is also regulated by the orexigenic neuropeptide Y (NPY) and kisspeptin, which relays both sex steroid and leptin signaling to GnRH neurons (49). In line with this, a high-fat diet increases hypothalamic *gnrh* expression in prepubescent gilts (50), where this treatment also advances puberty onset, a result observed also in female mice injected with leptin and in obese girls (50–52). Thus, this interplay between intrinsic (metabolic) and extrinsic (environmental) signals, sexual maturation and growth, likely represents an ancestral feature of the GnRH-like superfamily.

In this study, we show that the expression levels of *P. dumerilii gnrh-like* (*gnrhl*) preprohormones are increased in the brain of sexually mature individuals, after feeding, and in concert with the lunar cycle. Homozygous knockouts for *crz1/gnrhl1* are characterized by delayed maturation, reduced growth, and impaired regeneration, while food uptake appears unchanged. By a combination of proteomics and gene expression analyses, we identified a *lysosomal α-mannosidase*, 2 *α-glucosidases*, a *phosphoenolpyruvate carboxykinase*, and *glycogen synthase* as targets of CRZ1/GnRHL1. Taken together, our data suggest that *Platynereis* CRZ1/GnRHL1 signaling orchestrates growth and sexual maturation according to environmental conditions (nutrient availability and lunar phase), regulating glycoprotein turnover and energy homeostasis.

## Results

### *gnrh*-Like Preprohormones Are Expressed in Different Neuronal Clusters in the Brain of Adult Worms and Activate *Platynereis* GnRH-Like Receptors.

Four preprohormones of the *gnrh* superfamily were identified in *Platynereis* by a combination of transcriptomics, bioinformatics, and mass spectrometry analyses (refs. 53 and 54 and this study). Despite different similarities between the predicted prohormones and members of the different GnRH subfamilies from other bilaterians (53, 54), molecular phylogenetic analyses based on their amino acid sequence alone do not provide sufficient support for a decisive split into CRZ and AKH/GnRH subgroups (*SI Appendix*, Figs. S1 and S2*A*). To faithfully reflect this lack of unambiguous phylogenetic classification, we here adopt a dual nomenclature for these ligands, combining a general GnRH-like name (GnRHL1-4) with an additional name reflecting reliable deorphanization-based assignment to one of the established receptor orthology groups (13). This nomenclature reassigns the ligand previously called AKH1 (53) to GnRHL3 (see below) and renames AKH2 (53) to *Platynereis* GnRH2/GnRHL4 (please see *SI Appendix*, Table S1, for a synopsis of nomenclature and alternative proposals). In our sequence analysis, we found no conservation at the level of the C-terminal portion of propeptides, which in vertebrates contains the GnRH-associated peptide (GAP) (55, 56) (*SI Appendix*, Fig. S1).

As a first assessment if the GnRH superfamily system could be involved in adult biology, we performed whole-mount RNA in situ hybridization with the respective riboprobes on head samples of mature male and female individuals. These experiments allowed us to identify distinct neuronal clusters expressing 3 of the 4 *gnrh-like* genes (*SI Appendix*, Fig. S2 *B*–*D*). Whereas *crz2/gnrhl2* is also detectable in the brain by qRT-PCR (see expression analysis in Fig. 2), we could not determine a robust expression pattern, probably because of low expression levels. *Crz1*/*gnrhl1* expression was confined to 3 clusters located along the anteroposterior axis in the anterior part of the brain, at the level of the commissure between the anterior and posterior eyes (*SI Appendix*, Fig. S2*B*). The *gnrhl3* gene was mainly expressed in symmetrical patches in the medial forebrain domains expressing core circadian clock genes (8) (*SI Appendix*, Fig. S2*C*). Finally, *gnrh2*/*gnrhl4* localized in a deep single cluster between the 2 circadian domains as well as in close proximity to the anterior eyes (*SI Appendix*, Fig. S2*D*). These different expression patterns of *gnrh-like* genes likely reflect their contribution to distinct regulatory networks.

**Fig. 1.**
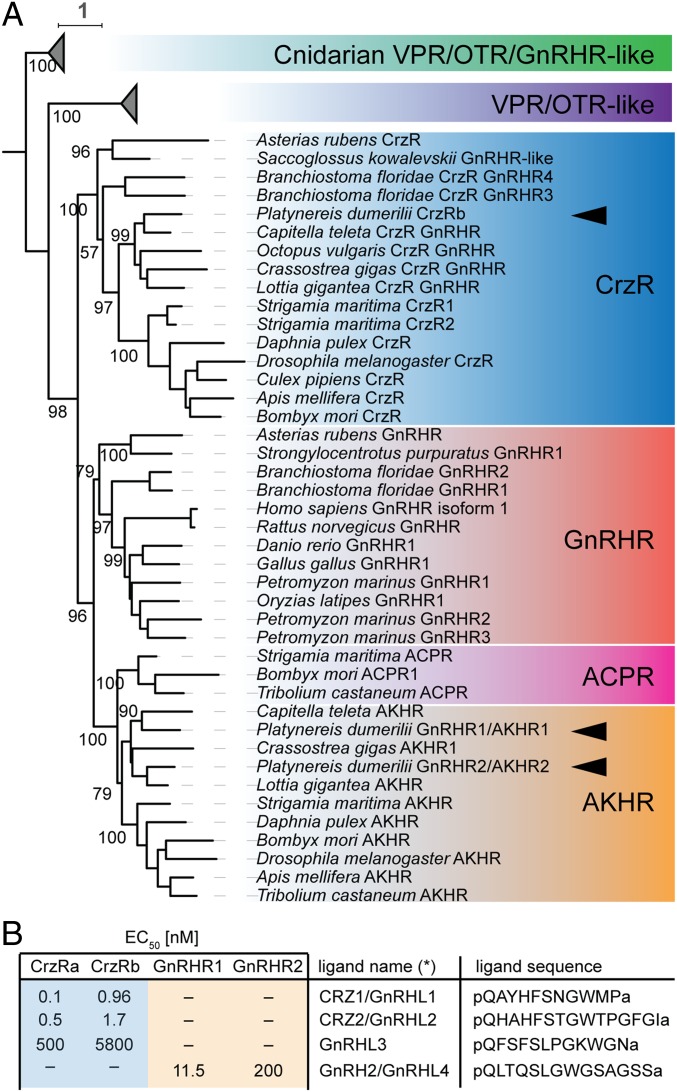
*P. dumerilii* possesses both CrzR and AKHR receptor orthologs that are differentially activated by 4 GnRH superfamily ligands. (*A*) Maximum likelihood phylogeny supporting the assignment of the 3 *Platynereis* receptors (arrowheads) of the GnRHR superfamily as members of the CrzR and AKHR subfamilies. Non-*Platynereis* sequences were retrieved from ref. 87 and complemented from the National Center for Biotechnology Information sequence repository. Bootstrap values, where not shown, are ≥55. (*B*) Receptor–ligand interactions as determined by systematic calcium mobilization assays performed with all receptor–ligand combinations. The table lists half maximal effective concentrations (EC_50_ values) for significant interactions. See *SI Appendix*, Fig. S3, for individual analyses.

A GnRH-superfamily receptor with clear orthology to the Corazonin Receptor subfamily (CrzR) was identified and shown to interact with both CRZ1 and CRZ2 (54). Systematic analyses of head-specific transcriptomes (57) allowed us to identify 3 additional receptors that belong to the GnRH receptor superfamily. Sequence analysis revealed that one of these was a different isoform of CrzR, in which 33 bp were missing from the 5′ end of the third exon, likely generated by the use of an alternative splice acceptor site (in the following, this isoform is referred to as CrzRa, to distinguish from the longer CrzRb variant). Molecular phylogenetic analyses confirmed the clustering of *Platynereis* CrzR with other CrzRs, while the other 2 newly identified GnRHRs clustered robustly within the AKHR group (Fig. 1*A*). Whereas this phylogeny suggests that the split in AKHR and ACPR families preceded the diversification of protostomes (implying a secondary loss of ACP signaling in lophotrochozoans), we adapt the suggestion of Zandawala et al. (13) to use a GnRH nomenclature for lophotrochozoans and hence refer to these receptors as GnRHR1/AKHR1 and GnRHR2/AKHR2. Finally, all cnidarian sequences retrieved by searches with either GnRHR-like or VPR/OTR-like receptors (the most closely related group to all GnRHRs; ref. 58) clustered together as outgroup to the represented bilaterian GnRHR/VPR/OTR-like receptors (Fig. 1*A*), consistent with the notion that these receptor families diverged only after bilaterian origin (58).

We next took advantage of a cell culture-based calcium mobilization assay (59) for assessing the ligand–receptor interactions of these receptors. This assay allowed us to deorphanize the newly discovered GnRHR1/AKHR1 and GnRHR2/AKHR2, and determine that they were specifically activated by GnRH2/GnRHL4 (Fig. 1*B* and *SI Appendix*, Fig. S3 *A* and *B*). Given that a receptor for GnRHL3 was still missing, we tested ligand–receptor interactions of all identified *Platynereis* GnRH-like receptors. Both CrzRa and CrzRb were activated by CRZ1/GnRHL1 and CRZ2/GnRHL2 (Fig. 1*B* and *SI Appendix*, Fig. S3 *C* and *D*; see also ref. 54) and, at higher concentration, also by GnRHL3 (Fig. 1*B* and *SI Appendix*, Fig. S3 *C* and *D*). As we presently cannot exclude the existence of an additional GnRH-like receptor in *Platynereis* that might be activated by GnRHL3 at lower concentrations, we here use the more generic name (GnRHL3) for this ligand.

In conclusion, molecular and functional analyses indicate that the GnRH-like system of *Platynereis* is complex: 1) a set of diversified ligands is expressed in distinct neuronal clusters in the brain and 2) 3 out of 4 ligands exclusively activate the same Corazonin-type receptor. These 2 apparently conflicting features might underline the existence of partially overlapping functions, yet coordinated by distinct neuronal networks.

### Transcripts of GnRH-Like Preprohormones Are Up-Regulated in Mature Animals, After Feeding and in Concert with the Lunar Cycle.

**Fig. 2.**
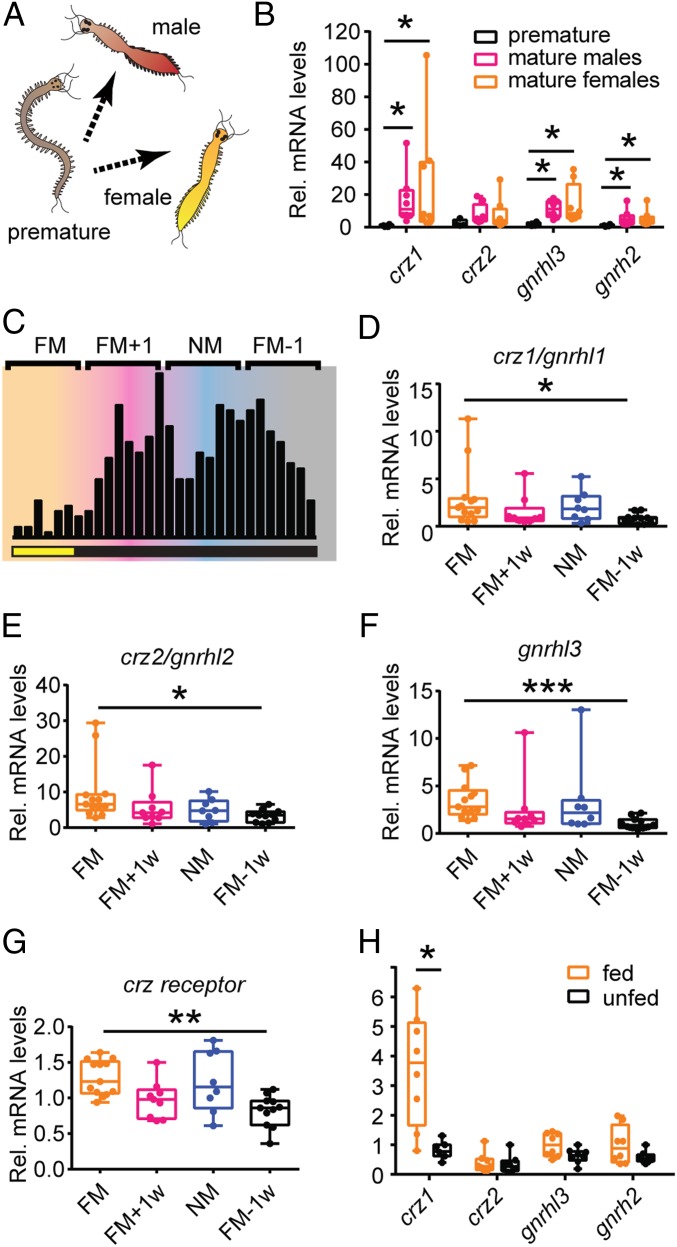
Transcript levels of *Platynereis gnrh-like* preprohormones are regulated by maturation, lunar phase, and food intake. (*A*) Schemes of premature worms, mature males, and mature females. (*B*) Relative expression levels of *gnrh-like* genes in premature (black), mature male (magenta), and mature female (orange) worm heads. *n* = 4 to 8 biological replicates. Statistical significance was tested using Kruskal–Wallis test followed by a Dunn’s multiple comparison test to adjust the *P* value for multiple testing. *P < 0.05. (*C*) Scheme showing the different sampling time points across the monthly lunar cycle (FM, full moon; FM+1, week after full moon; NM, new moon; FM-1, week preceding full moon). During the FM period, animals were exposed to 6 nights of a dimmer nocturnal light mimicking moonlight (lower bar). Black vertical bars schematize numbers of spawning wild-type animals (PIN strain) across a lunar month. Animals have been sampled between ZT7-9 in the days corresponding to the middle of the lunar phase/week. (*D*–*G*) *Crz1/gnrhl1*, *crz2/gnrhl2*, *gnrhl3*, and *crzr* are significantly up-regulated in the heads of late premature worms sampled during FM compared to animals collected in week FM − 1. *n* = 8 to 13 biological replicates. Statistical significance was tested using Kruskal–Wallis test followed by a Dunn’s multiple comparison test to adjust the *P* value for multiple testing. *P < 0.05; **P < 0.01; ***P < 0.001. (*H*) *gnrh-like* gene expression in the head of fed (spinach) or unfed (control) premature worms. *Crz1/gnrhl1* is significantly more abundant in the heads of fed animals compared to unfed controls. *gnrhl3* and *gnrh2/gnrhl4* show only a mild trend. *n* = 7 to 8 biological replicates. Statistical significance was tested using Mann–Whitney *U* test. *P < 0.05. For all experiments, 4 to 5 heads were pooled for single biological replicates. Relative expression was calculated using *cdc5* as reference gene, and data were normalized to the expression of premature worms (*B*), premature worms sampled during NM (*D*–*G*), and unfed worms (*H*).

In order to obtain further insight into the potential role of GnRH-like signaling in the bristleworm, we next investigated how the respective GnRH-like ligands were regulated on transcript level. In vertebrates and in several invertebrate taxa, GnRH-like preprohormones regulate sexual maturation and other aspects of reproduction. Thus, we wondered whether also in *Platynereis* these preprohormones can play a similar role. An increase in the expression of *gnrh-like* genes in sexually mature animals might suggest their involvement in the regulation of processes that directly (maturation or emission of gametes and mating) or indirectly (metabolic homeostasis and energy allocation) link to reproduction. Indeed, we found *crz1*/*gnrhl1*, *gnrhl3*, and *gnrh2*/*gnrhl4* significantly up-regulated in sexually mature animals (both males and females) compared to premature individuals (Fig. 2 *A* and *B*).

As mentioned, an aspect that is still poorly explored concerns the role that GnRH-like signaling plays in species that regulate their reproduction according to the lunar cycle. Circalunar and circasemilunar reproductive rhythms are widespread in marine animals (5) and likely evolutionarily ancient, yet insights about the underlying endocrine effectors are scarce (57, 60, 61). To assess whether 1) the expression of *gnrh-like* genes was regulated in phase with lunar cycle and 2) an increase in their expression might contribute to time-specific maturation patterns, we decided to analyze *gnrh-like* transcript levels in late premature worm heads sampled in each of the 4 wk of the lunar month (full moon [FM], FM + 1 wk, new moon [NM], and FM − 1 wk). Animals were exposed to a regular LD16:8 regime, and for 6 nights every month also to a dimmer nocturnal light mimicking moonlight (see ref. 8), and were fed 3 to 5 d prior the sampling. We found *gnrh-like* genes up-regulated during FM, compared to the preceding (*crz1*/*gnrhl1*, *crz2*/*gnrhl2*, and *gnrhl3*) or following (*gnrh2*/*gnrhl4*) lunar phases (FM − 1 wk and FM + 1 wk, respectively) (Fig. 2 *C*–*F* and *SI Appendix*, Fig. S4*A*). Interestingly, all of the genes encoding GnRH-like peptides capable of activating CrzR showed a significant increase in their expression levels in FM compared to FM − 1. We therefore also analyzed *crzr* transcript levels and found a consistent up-regulation in the transition from FM − 1 wk to FM (Fig. 2*G*), suggesting a synchronous strengthening in CrzR signaling during this phase. These data show that the *gnrh-like* gene expression in *Platynereis* is regulated in concert with the lunar cycle and indicate a possible contribution of GnRH-like signaling to the timing of sexual maturation.

Bristleworms metamorphose as the last step of their sexual maturation. This process is characterized by a stop of feeding, the development of secondary sexual characters, and final gonadal maturation. In addition to the lunar phase, the timing of this process is also regulated by metabolic cues as worms do not trigger this developmental transition before they attain a minimal size/weight. Given the substantial morphological and physiological changes that occur during this developmental transition under the absence of feeding, metamorphosis requires a sustained energy availability. For these reasons, we investigated whether in premature worms the expression of *gnrh-like* genes was affected by conditions strongly impacting on metabolism and energy availability, such as feeding and fasting. Two groups of animals were starved for 12 d, and subsequently, only 1 was fed with spinach for 24 h, prior to sampling. We found *crz1*/*gnrhl1* significantly up-regulated in the heads of postfed animals compared to unfed controls (Fig. 2*H*), suggesting the potential involvement of this gene in the regulation of feeding and/or metabolism.

Taken together, these results underline the potential relevance of GnRH-like signaling in the integration of key developmental, environmental, and metabolic cues to regulate maturation in *Platynereis*.

### Generation of *crz1/gnrhl1* Knockout Worms via Targeted Genome Editing.

**Fig. 3.**
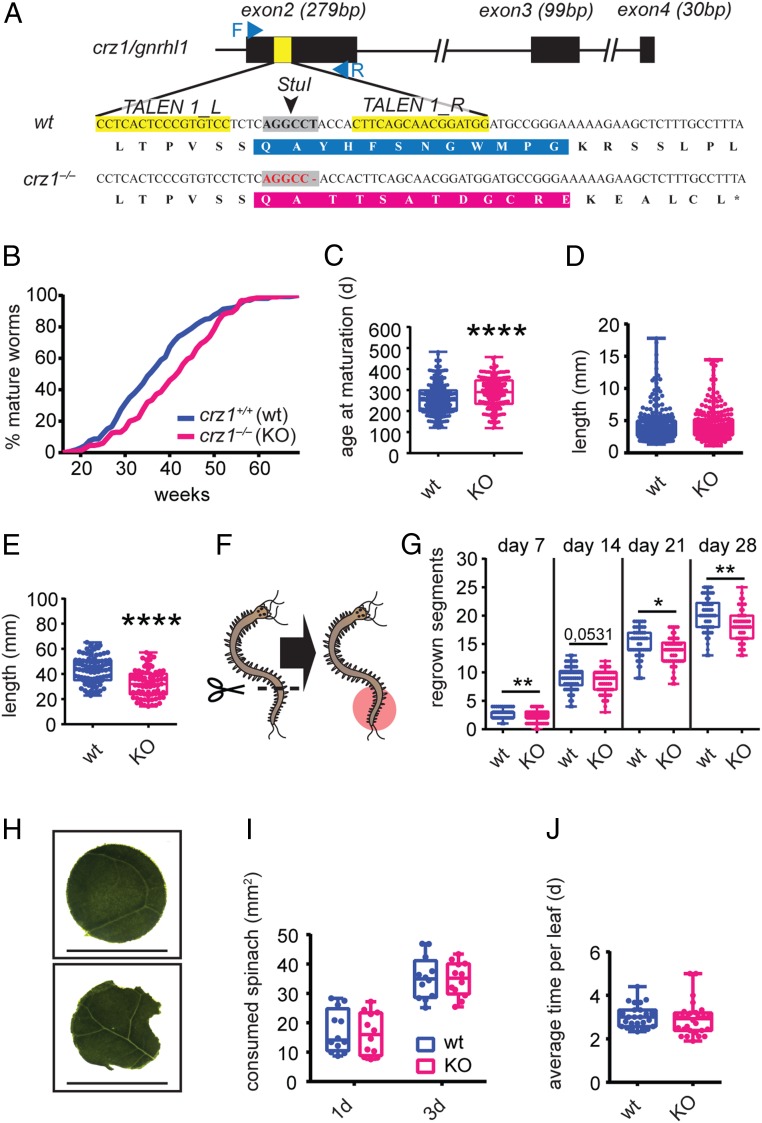
Engineered *crz1* mutants exhibit maturation delay, dampened growth rate, and posterior regeneration, while food intake is unaffected. (*A*) Schematized *crz1/gnrhl1* locus highlighting the site in the first coding exon (exon 2) selected for targeted mutagenesis and the alignment of wild-type and *crz1/gnrhl1* mutant gene sequences (*Bottom*). Black rectangles represent exons, and yellow sections highlight the binding sites for TALENs. Blue arrows schematize primers used for genotyping, and gray areas correspond to the restriction site around which TALENs were designed. In the DNA sequence alignment, the resulting amino acid sequences of wild-type peptide (blue) and the corresponding aberrant peptide (magenta) are boxed. (*B* and *C*) In *crz1* mutants (KO), maturation is delayed compared to wild-type siblings. Data are presented as cumulative curves based on the percentage of mature animals over time (weeks) (*B*), as well as plotting the age at which worms reached maturation (*C*). Wild types are shown in blue, and mutants are shown in magenta. *n* ≥ 213. Statistical significance was tested using *t* test. ****P < 0.0001. (*D* and *E*) *crz1* mutants (KO) exhibit a reduction in growth rate. (*D*) Length in mm of 2-mo-old young worms does not differ between *crz1*^*−/−*^ and ^*+/+*^. *n* ≥ 232. (*E*) Length of 5-mo-old premature *crz1*^*−/−*^ and ^*+/+*^ worms. Age-matched mutant worms are significantly smaller compared to wild-type controls. *n* ≥ 75. Statistical significance was tested using *t* test. ****P < 0.0001. (*F* and *G*) Posterior regeneration is dampened in *crz1* mutants (KO). (*F*) Schematic representation of the experimental design. Fifteen segments have been amputated from age-matched ∼50-segment-long premature *crz1*^*−/−*^ and ^*+/+*^ worms, and regenerated segments (red area) have been counted weekly for 4 wk. (*G*) Mutants regenerate a reduced number of segments per time unit (week) compared to wild types. Data are shown as cumulative number of regenerated segments for all of the 4 wk. *n* ≥ 48. Statistical significance was tested using *t* test. *P < 0.05; **P < 0.01. (*H*–*J*) Food intake is not affected in *crz1* mutants (KO). (*H*) Representative spinach circle provided in the feeding experiments and representative spinach circles after a 1-d experiment. (Scale bar, 18 mm.) The eaten area was calculated subtracting the residual leaf area (*Bottom*) from the averaged area of 6 control leaves (*Top*). (*I*) Eaten leaf area reported for *crz1*^*−/−*^ and ^*+/+*^ worms in the 2 time points (after 1 and 3 d). *n* = 12. (*J*) Food intake rate is not affected in *crz1*^*−/−*^ mutants. Data are reported as average time (days) required to eat completely a single spinach circle. *n* ≥ 25.

In order to characterize the function of the GnRH system of *Platynereis*, we next decided to generate a knockout strain. As a target, we selected *Platynereis crz1/gnrhl1* as this was the *gnrh-like* gene that showed the most remarkable regulation in the tested conditions (Fig. 2 *B* and *H*). Specifically, we targeted TALENs against the first coding exon of *crz1/gnrhl1* and injected them into *Platynereis* zygotes following established procedures (11). Injected animals were raised till maturation and then outcrossed to wild-type individuals. Progeny from several crosses were screened for inherited mutations. We identified worms carrying a 1-bp deletion in the region encoding the mature peptide. This mutation caused a frame shift predicted to produce an aberrant mature peptide, as well as a premature stop codon after 16 amino acids (Fig. 3*A*). By incrossing heterozygous individuals, we managed to establish a homozygous *crz1/gnrhl1*^*Δ1/Δ1*^ strain (hereafter referred to as *crz1*^*−/−*^) that we compared to respective *crz1*^*+/+*^ controls.

### *Crz1* Knockout Animals Exhibit Delayed Maturation, Reduced Growth, and Regeneration Impairment but No Effects on Food Uptake.

The observed regulation of *crz1/gnrhl1* transcripts after feeding and in concert with the lunar phase, as well as in sexually mature animals, suggests a role in maturation, a process which requires the integration of both internal (metabolic) and external information. As *crz1*^*−/−*^ worms mature without obvious reproductive deficits or developmental abnormalities, we tested for an involvement in the regulation of maturation timing. We reared *crz1*^*−/−*^ and *crz1*^*+/+*^ animals in density-controlled conditions and with the same feeding regime, scoring the day in which they, after the completion of metamorphosis, attain sexual maturity and engage in a nuptial dance that culminates in spawning. We found that mutants markedly delayed this event (∼5 wk) (Fig. 3 *B* and *C* and *SI Appendix*, Fig. S4 *B*–*E*). Given its extent, we excluded that such a delay was solely due to a slower progression through metamorphosis, a process that is completed on average in 7 to 8 d. We therefore assessed whether the maturation delay could be caused by a reduction in the growth rate and a resulting later attainment of the critical size for developmental progression. To test this hypothesis, we first measured the length of 2-mo-old *crz1*^*−/−*^ and *crz1*^*+/+*^ young worms reared in similar density conditions, observing no difference (Fig. 3*D*). Next, we isolated mutant and wild-type young immature worms in separate boxes with the same final density and fed them with identical amounts of food. After 3 additional months (5 mo after fertilization), we measured both the total length of the animals and the number of their segments. Now, *crz1*^*−/−*^ worms had a significant reduction in both values compared to controls (Fig. 3*E* and *SI Appendix*, Fig. S4*F*). To independently test whether *crz1/gnrhl1* was involved in the regulation of posterior growth, we assessed caudal regeneration, a process that recapitulates many aspects of normal posterior elongation in a faster manner (62, 63). For these assays, we amputated the last 15 segments from ∼50-segment-long premature worms and subsequently counted the number of regrown segments for the following 4 wk (Fig. 3*F*). *Crz1*^*−/−*^ worms exhibited a significant reduction of regenerated segments in weeks 1, 3, and 4 (week 2, *P* = 0.0531) after the injury (Fig. 3*G*).

Both the maturation delay and the attenuated growth/regeneration in postlarval *crz1*^*−/−*^ worms could be explained by lower food intake. As outlined above, *crz1/gnrhl1* itself showed a remarkable overexpression when food is available (Fig. 2*H*), suggesting a possible orexigenic function. To test this hypothesis, we designed 2 feeding assays for *Platynereis* postlarval stages, adapting an experimental paradigm used for the snail, *Lymnaea stagnalis* (64). In the first assay, premature worms were subjected to 12 d of fasting and afterward were individually isolated and fed with a circular disk of spinach each (Fig. 3*H*). At 2 time points (after 1 and 3 d), we quantified the consumed amount of spinach by measuring the remaining area in each disk. In the second assay, we measured the food intake rate (number of eaten leaves/time) during a total period of 5 wk. In this case, isolated premature worms were subjected to short starvation (5 d) and were subsequently fed with circular spinach pieces that were freshly provided as soon as the previous one was entirely eaten. In both of these approaches, *crz1*^*−/−*^ and wild-type animals did not exhibit any significant differences in food intake (Fig. 3 *I* and *J*). We therefore dismissed the hypothesis that the phenotype of *crz1*^*−/−*^ animals was due to an orexigenic function of CRZ1/GnRHL1 in *Platynereis*, in favor of a contribution of this gene to energy homeostasis.

### CRZ1/GnRHL1 Regulates the Expression of Key Enzymes for Glycoprotein and Carbohydrate Metabolism.

We next decided to identify potential target genes that could explain the observed phenotype of *crz1* knockouts. In a candidate-driven approach, we identified *Platynereis* orthologs of the vertebrate gonadotropins (termed GPA and GPB; *SI Appendix*, Fig. S5*A*). Both *gpa* and *gpb* genes exhibited different transcript levels in mature males and female worms, but neither this differential expression nor the expression in premature animals appeared to be changed in *crz1*^*−/−*^ animals (*SI Appendix*, Fig. S5 *B*–*E*). In complementation to the candidate-driven approach, we therefore performed an unbiased screen for potential targets, adopting a combination of protein mass spectrometry and gene expression analysis. We first performed a quantitative proteomic analysis on heads of sexually mature males and females from both genotypes (*crz1*^*−/−*^ and *crz1*^*+/+*^). This choice was based on several reasons: sexually mature worms showed the highest absolute expression levels of *crz1/gnrhl1*, thereby making it more likely to observe dysregulation of target factors; moreover, mature animals represent the developmental stage at which a fine-tuned regulation of energy homeostasis is crucial. Indeed, sexually mature *P. dumerilii* worms commit to an “all in” metabolic performance to reproduce, engaging in an energy-demanding nuptial dance that ultimately leads them to death after spawning. Finally, mature worms do not feed, further reducing complexity of the analysis.

**Fig. 4.**
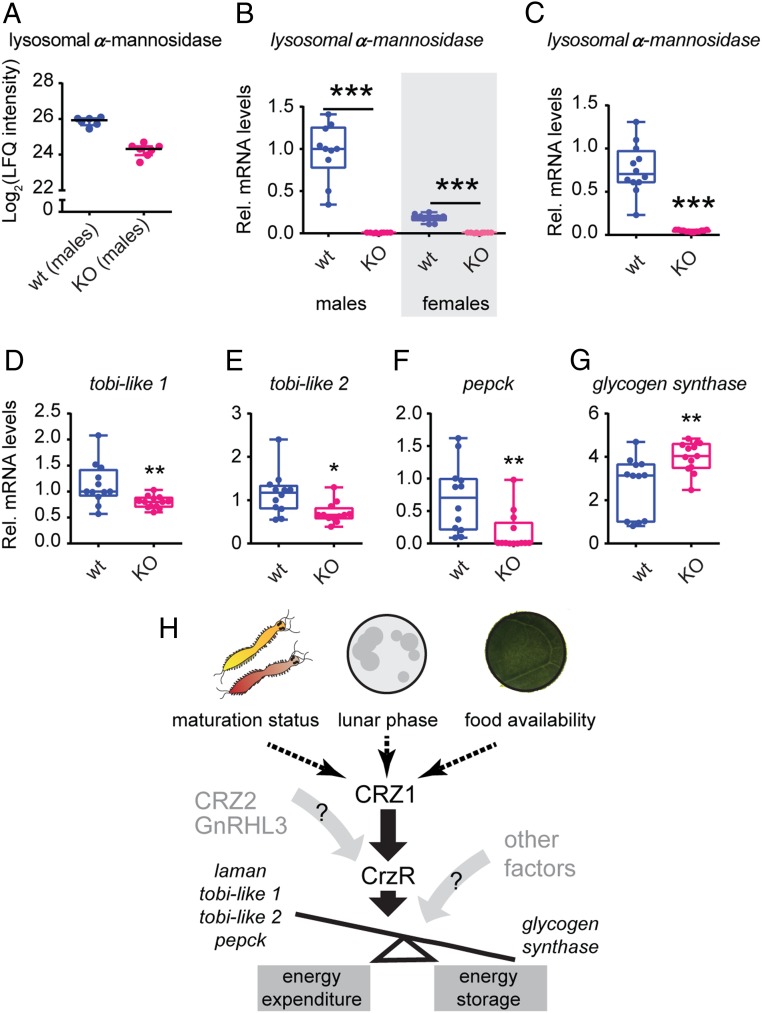
CRZ1/GnRHL1 contributes to glycoprotein turnover and carbohydrate homeostasis. (*A*) Down-regulation of lysosomal α-mannosidase in heads of *crz1*^*−/−*^ (KO) specimens (*Right*) compared to wild-type controls (*Left*). Plot extracted from proteomic comparison, represented as LFQ intensity. Lysosomal α-mannosidase is 1.6-fold enriched in the male ^+/+^ proteome compared to the ^−/−^ counterpart. *n* = 6 to 7. (*B* and *C*) Relative mRNA levels in metabolically active tissues (trunk) of the *lysosomal α-mannosidase* identified by mass spectrometry analysis. This gene was strongly down-regulated in both *crz1*^*−/−*^ (KO) mature males and females (*B*), as well as in *crz1*^*−/−*^ (KO) premature worms (*C*) compared to wild-type counterparts. (*D*–*G*) *crz1* knockout (KO) affects the expression of enzymes involved in glucose homeostasis at the transcript level. Relative expression levels of *tobi-like 1* (*D*) and *tobi-like 2* (*E*) *α-glucosidases* are significantly down-regulated in *crz1* mutants (KO), as well as the identified *pepck* (*F*). Inversely, mRNA levels of glycogen synthase were significantly up-regulated in *crz1* mutants (KO) (*G*). For all qRT-PCR experiments, 4 to 5 trunks were used for single biological replicate. Relative expression was calculated using *sams* as reference gene, and data were normalized to the expression of *crz1*^*+/+*^ (wt) controls. *n* = 10 to 13 biological replicates. Statistical significance was tested using Mann–Whitney *U* test. *P < 0.05; **P < 0.01; ***P < 0.001. (*H*) Graphical summary of the presented data and the proposed contribution of *Platynereis* CRZ1/GnRHL1 signaling to the regulation of the energy balance according to developmental and environmental cues and the coordination of processes such as developmental progression and growth.

We decided to focus our efforts initially on heads in order to avoid large proteomic biases from sperm- and egg-specific proteins present in the trunk of mature animals. To minimize variation, we collected all samples between ZT7 and ZT9, coherent with all other experiments. This approach allowed us to identify a *Platynereis* homolog of lysosomal α-mannosidase, which is significantly down-regulated in mutant males compared to their wild-type male siblings (Fig. 4*A* and *SI Appendix*, Fig. S4*G*). α-mannosidases are a class of ubiquitous enzymes responsible for the hydrolysis of single mannose residues and mainly known for their role in *N*-linked carbohydrate catabolism during glycoprotein turnover (65). In light of the possible link between *crz1/gnrhl1* signaling and catabolism, we wondered whether the identified α-mannosidase was also regulated in the trunk, where most metabolic processes take place. To bypass possible food contaminations, and as reliable proteomic analysis of *Platynereis* trunk are technically very challenging due to extremely resistant and active proteases, we focused these analyses on a qRT-PCR approach. When investigating *lysosomal α-mannosidase* expression in mature animals, we observed a marked down-regulation in both *crz1*^*−/−*^ male and female trunks compared to wild-type specimens (Fig. 4*B*). Likewise, when studying the expression of *lysosomal α-mannosidase* in fed premature worms (where feeding induced high levels of *crz1/gnrhl1*; Fig. 2*H*), *crz1*^*−/−*^ worms exhibited an almost complete down-regulation of *lysosomal α-mannosidase* when compared to wild-type counterparts (Fig. 4*C*). In both flies and humans, changes in expression and activity levels of α-mannosidases have been linked to impairments in insulin signaling and glucose metabolism (66–70). For instance, in *Drosophila*, the ablation of insulin-producing cells of fed flies increased the expression of a *lysosomal α-mannosidase* in concurrence with a down-regulation of an evolutionary conserved *α-glucosidase—target of brain insulin* (*tobi*) (66)—whose overexpression reduces body glycogen. Based on these findings, we extended our gene expression analysis to enzymes involved in glucose and glycogen metabolism, known to be regulated also at the transcript level. Also in this case, we focused on fed premature worm bodies, where also anabolic processes can be investigated. Using sequence-similarity and molecular phylogenetic approaches (*SI Appendix*, Fig. S7), we identified 2 *tobi-like α-glucosidases* (called hereafter *tobi-like 1* and *2*), as well as homologs of *phosphoenolpyruvate carboxykinase* (*pepck*, one of the key enzymes contributing to gluconeogenesis) and *glycogen synthase* in *Platynereis* published transcriptomes (53, 57). When we analyzed the transcript levels of these genes in *crz1*^*−/−*^ and wild-type worms, we observed a significant down-regulation of both *tobi-like 1* and *tobi-like 2*, as well as the identified *pepck*, whereas *glycogen synthase* was significantly up-regulated (Fig. 4 *D*–*G*). Taken together, these analyses identified *lysosomal α-mannosidase* and plausibly glycoprotein turnover as primary targets of CRZ1/GnRHL1 signaling in *Platynereis* and suggested the relevance of this hormone for the regulation of glucose homeostasis in the worm (Fig. 4*H*).

## Discussion

Animals promote developmental progression and reproductive events in favorable environmental conditions, integrating multiple extrinsic and intrinsic cues. However, it is still unclear how this complex orchestration occurs and what is the nature of the molecular switch in the brain that regulates energy expenditure accordingly. Here we show that all 4 identified *Platynereis* GnRH-like peptides (CRZ1/GnRHL1, CRZ2/GnRHL2, GnRHL3, and GnRH2/GnRHL4) activate GnRH-superfamily receptors in vitro, with results suggesting cross-activation at physiologically relevant concentrations. We find *gnrh-like* preprohormone transcripts not only up-regulated in the brains of sexually mature animals but also after feeding and in specific lunar phases. Homozygous knockout worms for *crz1/gnrhl1* (the most inducible *gnrh-like* preprohormone) exhibit a maturation delay, coupled with a decrease in growth rate and regeneration. As mutants show normal food intake compared to wild types, this points at alterations in the mechanisms governing energy homeostasis. Consistent with this, our combined proteomics and gene expression analyses identify changes in the levels of factors involved in glycoprotein and carbohydrate metabolism. Our data therefore imply *Platynereis crz1/gnrhl1* as a factor that helps to adjust energy balance in accordance with the metabolic demand that is dictated by environmental and developmental cues and thereby contributes to regulate sexual maturation and growth.

The *crz1/gnrhl1* mutant strain we generated represents one of the few mutants for *gnrh-like* genes in invertebrates, and provides information on the function of GnRH-like peptides outside insects. In addition, *P. dumerilii* has emerged as a useful lophotrochozoan reference model that allows cross-comparisons with other bilaterians in aspects such as sensory, hormonal and developmental biology (71–74). The phenotypic and molecular characterization of *crz1* mutants presented here might therefore offer insight into the functions of a hormone family whose members are present in several metazoan lineages.

A distinct molecular phenotype revealed by our mutant analysis is the regulation of *lysosomal α-mannosidase* by the *Platynereis* corazonin signaling system. Whereas we have not attempted to resolve the effects for cellular metabolism in the worms, insight from other systems strongly suggests that this down-regulation leads to unprocessed mannose-rich glycans and related impairment in glycoprotein turnover (see above and *SI Appendix* for published accounts on the effects of lysosomal α-mannosidase impairments). In parallel, we also reveal a significant up-regulation of *glycogen synthase*, whose experimental overexpression has been shown to cause an increase in glycogen levels in both isolated muscle fibers and mice (75, 76). In conjunction with the observed deregulation of other key enzymes for glucose metabolism in *crz1* mutants, our data therefore indicate that CRZ1/GnRHL1 impacts on *Platynereis* energy homeostasis, shifting the balance between catabolic and anabolic processes (Fig. 4*H*). Specifically, mutant animals likely have a more dampened ability in making glucose available for cellular processes. This link between corazonin signaling and carbohydrate metabolism provides a plausible explanation for the attenuated growth rate and regeneration potential in *crz1*^*−/−*^ worms.

One implication of this model is that the regulation of CRZ levels in wild-type animals reflects conditions under which there is a specific demand for regulating the mobilization of glucose. Our qRT-PCR data reveal an up-regulation of *crz1/gnrhl1* transcripts in sexually mature animals, fed worms, and specific lunar phases. Whereas these appear to be rather different conditions, energy mobilization indeed provides a common denominator: after feeding, the up-regulation of enzymes involved in breaking down glycans and adjusting glucose homeostasis may have a direct function to convert food into appropriate levels of energy and coordinate metabolic processes in a condition of high energy availability; in turn, in mature animals, the mobilization of (internal) energy stores would be relevant to fuel the terminal mass spawning event; finally, our finding that *gnrh-like* signaling is also regulated over the lunar cycle may reflect the timing of specific metabolic processes to provide the energy required at metamorphosis onset (also see below for the aspect of reproductive timing).

The focus on the metabolic consequences of CRZ/GnRHL provides an interesting perspective for the cross-comparison of corazonin signaling with other signaling systems. The proposed role of worm CRZ1 in promoting the expression of *α-glucosidases* and *pepck* is reminiscent of the action of the GnRH-like AKH peptide in flies but also the role of vertebrate glucagon, an independent factor that has been discussed as a functional analog of insect AKH signaling (38, 66, 77–79). Both of these can elicit increased glucose production by both glycogenolysis and gluconeogenesis. Thus, CRZ1/GnRHL1 seems to rely on a set of downstream effectors and mechanisms that are also employed by well-established regulators of carbohydrate catabolism. Our data are therefore consistent with the idea that energy homeostasis and allocation might be ancestral functions regulated by GnRH-like systems, even if the net effects on carbohydrate metabolism might differ between individual members (40). As reproductive events are often associated with the dedication of significant energy resources, such a link to energy homeostasis would provide a plausible explanation why members of the GnRH-like superfamily carry roles in reproduction or other life history events. Indeed, also in insects, CRZ signaling regulates both aspects of metabolism (40, 80) and reproduction (sperm transfer) (29, 81), as well as developmental timing (larval–pupal transition and pupariation) (82) (see *SI Appendix* for additional evidence for the role of CRZ/GnRH members in the regulation of life histories).

Due to its semelparous breeding strategy, *P. dumerilii* is a model system in which energy homeostasis has central relevance. The commitment of worms to reproduction is linked to a fundamental metabolic transition: while worms become anorexic, they rely entirely on the breakdown of energy stores and recycling in order to undergo profound morphological changes (metamorphosis) and complete gamete maturation. As the ultimate gamete release is synchronized with the lunar phase, the regulation of metamorphosis onset and energy homeostasis is an interesting entry point to get insight into the elusive molecular machinery involved in reproductive timing. On average, the completion of metamorphosis lasts 7 to 8 d. As laboratory worms avoid entering metamorphosis in the week preceding the artificial full moon stimulus (*SI Appendix*, Fig. S6 *A* and *E*), the number of spawning animals resulting from this regulation is lowest during the full moon phase (8, 11) (see also *SI Appendix*, Fig. S6 *B* and *D*). In light of these considerations, the significant difference of *crz1/gnrhl1* as well as *crzr* transcript levels in the week preceding the nocturnal light stimulus (FM − 1; lower expression) and the full moon week itself (FM; higher expression) is consistent with the idea that this regulation is part of the mechanism that directs gamete release to the week following the full moon period (FM + 1).

Despite the regulation of *crz1/gnrhl1* transcripts between different lunar phases, *crz1*^−/−^ worms exhibit an obvious difference neither in their onset of metamorphosis (cf. *SI Appendix*, Fig. S6 *A*, *C*, *E*, and *G*) nor in their lunar spawning pattern (cf. *SI Appendix*, Fig. S6 *B*, *D*, *F*, and *H*). A possible explanation for the apparent lack of a strong rhythmicity phenotype is that the system may contain redundancy: indeed, our results not only indicate that the CrzR receptor bundles inputs from 2 ligands (CRZ2 and GnRHL3) besides CRZ1, but both of them also share a similar regulation profile across the lunar month. A prediction from this hypothesis is that mutation of the corazonin receptor gene (*crzr*) might yield stronger phenotypes than a single ligand mutation such as the *crz1* knockout. Furthermore, we note that the current paradigm for circalunar rhythmicity (number of worms spawned per day) yields relatively broad peaks, likely because entrainment conditions are not as precise as natural stimuli (6). This might prevent subtle phenotypes from being detected.

Taken together, the evidence reported here suggests *Platynereis* CRZ1 as one of the factors that coordinate the response of the animal to specific energy demands dictated by both environmental and developmental cues, in order to orchestrate growth and maturation.

## Methods

Detailed methods on the following subjects are available in *SI Appendix*, *SI Methods*: worm culture and phenotypic analysis, qRT-PCR, tissue lysis and proteolytic digestion, LC-MS/MS analysis, proteome data analysis, phylogenetic studies, and sequence analyses.

### Worm Culture and Phenotypic Analysis.

*Platynereis* cultures were maintained at 18 °C (LD16:8) according to standard protocols as described previously (83). All animal work was conducted according to Austrian and European guidelines for animal research. Details concerning phenotypic analysis are provided in *SI Appendix*.

### In Situ Hybridization.

*Platynereis* in situ hybridization on heads from sexually mature worms was performed in accordance with previous experiments (8). Riboprobes for *Platynereis crz1/gnrhl1*, *gnrhl3*, and *gnrh2/gnrhl4* were generated by subcloning the respective cDNA sequences into pJET1.2 vector and transcribing antisense riboprobes according to established protocols (8, 9). Stained animals were mounted in DABCO-glycerol. Pictures were taken on a Zeiss Axioplan4 microscope using 10× or 20× Plan-Neofluar objective (dry) or 40× Neofluar objective (oil immersion). Images were recorded with Zeiss AxioCam MRC 5 camera using Zeiss ZEN AxioVison software.

### Deorphanization Assay.

CrzRb was identified and initially deorphanized in ref. 54. GnRHR1/AKHR1 and GnRHR2/AKHR2, as well as the second isoform of CrzR (CrzRa) were identified using an in silico approach and confirmed by cloning and sequencing. To facilitate deorphanization, cDNA of these receptors was amplified from laboratory *Platynereis* strains and cloned into the pcDNA3.1 (+) vector. The respective vectors were expressed by transient transfection in Chinese Hamster Ovary (CHO) cells which stably express apo-aequorin and the human Gα_16_ subunit. The G_α16_ protein couples to most GPCRs and directs signaling to the Ca^2+^ pathway regardless of their endogenous G protein coupling, which allows monitoring GPCR activation by measuring intracellular Ca^2+^ levels (for further details, see ref. 59). Several peptide concentrations were tested for each synthetic peptide and its corresponding activated receptor. Additionally, cells for negative control experiments were transfected with empty pcDNA, and ATP was used as a positive control. Receptor activation was measured by monitoring calcium responses for 30 s on a Mithras LB 940 luminometer (Berthold Technologies). Percentage activation values of receptors were plotted for each receptor, and half maximal effective concentrations (EC_50_ value) were calculated using a computerized nonlinear regression analysis with a sigmoidal dose–response equation (GraphPad PRISM software). The dose–response curves arise from at least 4 replicates conducted in at least 2 independent experiments. All of the synthetic peptides were synthesized by the Mass Spectrometry Facility of the Institute for Molecular Pathology of the University of Vienna, and the purity of these peptides was checked by matrix-assisted laser desorption/ionization time-of-flight mass spectrometry (MALDI-TOF-MS) by the Chemistry Department of the University of Vienna.

### qRT-PCR.

Worms from the PIN wild-type strain were used to assess expression levels of *gnrh-like* genes in different contexts (Fig. 2 *B* and *D*–*H*). Worm heads were quickly decapitated between ZT7 and ZT9, snap-frozen in liquid nitrogen, and stored at −80 °C. For each biological replicate, mRNA was extracted from 4 to 5 worm heads or trunks (all of the body parts remaining after decapitation), using RNeasy Mini Kit (QIAGEN), and reverse transcription was carried out using QuantiTect Reverse Transcription kit (QIAGEN). qRT-PCR was performed using PowerUp SYBRGreen Master Mix (Applied Biosystems) and QuantStudio 3 System (Applied Biosystems). mRNA levels were normalized to the levels of *cdc5* for heads (8) and *sams* (57, 84) for trunks, and relative quantification was performed by using the 2^−ΔΔCT^ method. Results were analyzed using Mann–Whitney *U* test or Kruskal–Wallis test with Dunn’s post hoc test to correct for multiplicity when required: *P < 0.05; **P < 0.01; ***P < 0.001. For primers list, see *SI Appendix*.

### Generation and Genotyping of *crz1* Knockout Worms.

TALENs design and preparation were carried out as previously described (11). We targeted exon 2 in the *crz1/gnrhl1* locus, with the TALENs recognition sites (capitalized) flanking an endogenous StuI site (underlined): 5′-CCTCACTCCCGTGTCCtctcaggcctaccacTTCAGCAACGGATGG-3′. The *crz1*^*−/−*^*/gnrhl1*^*−/−*^ strain was generated from a single founder, carrying a 1-bp deletion in the region encoding the CRZ1/GnRHL1 mature peptide. This resulted in a frameshift mutation that abrogated almost the entire mature peptide amino acid sequence and also introduced a premature stop codon after 16 amino acids (Fig. 3*A*). To genotype worms, the region encompassing TALENs-targeted site was amplified (primers F: 5′-GAACTGTTCGTGGGTGTCCT-3′, R: 5′-GTTCGGCAGAAACTGAGGTC-3′) and the restriction digestion performed using *StuI* (New England Biolabs).

### Tissue Lysis and Proteolytic Digestion and Proteome Data Analysis.

Tissue lysis and subcellular fractionation were performed applying a previously established protocol (85). For details about LC-MS/MS analysis and proteome data analysis, see *SI Appendix*. The MaxQuant software (version 1.6.0.1), including the Andromeda search engine, was used for data analysis (86).

### Statistical Analysis.

Statistical analysis has been performed using R Studio and Graph Pad Prism 8 software.

### Data Availability.

Sequence identifiers for genes described in this study are listed in *SI Appendix*, Tables S1–S3. Plasmids for cloned genes (*SI Appendix*, Tables S1 and S2) as well as worm strains described in this study are available upon request from F.R.

## Supplementary Material

Supplementary File
