# Supplementary material for: Corazonin signaling integrates energy homeostasis and lunar phase to regulate aspects of growth and sexual maturation in Platynereis
Source: Proc Natl Acad Sci U S A. 2019 Dec 16;117(2):1097–106. doi: 10.1073/pnas.1910262116 (PMC6969523; doi:10.1073/pnas.1910262116)
Supplement: Supplementary File [file pnas.1910262116.sapp.pdf]

PNAS
www.pnas.org

Supplementary Information for

Corazonin signaling integrates energy homeostasis and lunar phase to regulate aspects of growth and sexual maturation in *Platynereis*

Gabriele Andreatta, Caroline Broyart, Charline Borghgraef, Karim Vadiwala, Vitaly Kozin, Alessandra Polo, Andrea Bileck, Isabel Beets, Liliane Schoofs, Christopher Gerner and Florian Raible

Corresponding authors: Gabriele Andreatta, Florian Raible

E-mail: gabriele.andreatta@univie.ac.at, florian.raible@univie.ac.at

This PDF includes:

Supplementary Text

Figures S1-S7

Tables S1-S3

SI References

www.pnas.org/cgi/doi/10.1073/pnas.1910262116

Supplementary Text

Platynereis dumerilii GnRH superfamily ligands

The four peptides tested in our deorphanisation assay are identical to peptides predicted in the study by Conzelmann et al. (2013) (ref. 1), but their nomenclature has meanwhile been revised, and our own deorphanisation work suggests a different classification for one of these (GnRHL3, previously referred to as AKH1/AKH). No final consensus has been reached about the nomenclature of components of the GnRH-like system (see refs. 2,3). As outlined in the main text, our naming of ligands follows the basic suggestions by Zandawala et al. (2), while also using a more generic naming system (GnRH-like/GnRHL) that is more faithful with respect to the missing certainty about ligand orthology, and also appears most appropriate for GnRHL3, given that the EC50 levels for the GnRHL3-CrzR activation assay are significantly higher than for the other CRZ ligands. For clarification, **Table S1** provides an overview of the investigated peptides, alternative names occurring in the literature, and the corresponding Genbank identifiers.

Platynereis dumerilii GnRH superfamily receptors

In contrast to the GnRH superfamily ligands, orthology was more reliable to establish for the GnRH superfamily receptors (see **Fig.1**). For referring to the *Platynereis* receptors clustering along with the insect AKH receptors, we adapted the GnRH receptor nomenclature suggested by Zandawala et al. (2). **Table S2** provides an overview over the receptor sequences described in the study.

Additional *Platynereis dumerilii* genes

Table S3 lists the additional genes whose expression was analysed in this study. For the five metabolic enzymes (lysosomal α-mannosidase, tobi-like 1 and 2, phosphoenolpyruvate carboxykinase, and glycogen synthase), phylogenetic analyses are included in **Fig. S7**. The analysis of glycoprotein hormone alpha and beta is included in **Fig. S5** and further discussed below.

Adult *Platynereis* heads express orthologs of the vertebrate glycoprotein hormone system

In vertebrates, GnRH controls not only the pulsatile secretion, but also the expression of pituitary gonadotropins (6–10). Interestingly, a GnRH-like peptide from *Octopus vulgaris* that acts through a Corazonin-type receptor (11) stimulates LH-release from cultured quail pituitary cells (12). The vertebrate gonadotropins (LH and FSH), as well as the metabolic-related factors thyroid-stimulating hormone (TSH) and thyrostimulin (TS), are glycoprotein hormone (GPH) heterodimers (13, 14). The α and β subunits of GPHs form distinct groups that are found across all bilaterians, suggesting that a GPH-like system already existed in early animal ancestors (15, 16). Given that also GnRH-like systems are part of this ancestral repertoire, we investigated if a functional relationship between GnRH-like peptides and GPHs existed in the bristleworm.

In *Platynereis dumerilii*, a GPHβ-like subunit is referred to as GPB (1). Our independent searches yielded not only this subunit, but also a partial sequence encoding a putative glycoprotein α-like subunit that we named GPA (**Table S3**). In line with previous phylogenetic analyses (15–17), GPA and GPB cluster with GPHα and GPHβ subunits from other invertebrates, respectively (Fig. S5A). Moreover, our analysis suggests that the common bilaterian ancestor possessed single GPA and GPB subunits, and that these diversified further in the evolutionary lineage leading to vertebrates, giving rise to (i) vertebrate thyrostimulin (GPHα2 and GPHβ5) and (ii) vertebrate gonadotropin/thyroid hormone (GPHα1 and FSHβ/LHβ/TSHβ) systems, a diversification that is likely linked to the whole genome duplications that occurred in vertebrate evolution. We therefore refer to the invertebrate glycoprotein hormone subunits simply as GPA and GPB, respectively. To investigate whether the GnRH-like system in *Platynereis* could also be involved in the regulation of vertebrate glycoprotein hormone orthologs, we analyzed the expression levels of both *gpa* and *gpb* in sexually mature and fed premature worms, two conditions that we knew to exhibit higher *crz1/gnrhl1* levels (see Fig. 2B and H). When comparing *crz1-/-* samples with their respective wild-type counterparts, transcript levels for both *gpa* and *gpb*

did not significantly differ in both of these settings (Fig. S5B-E). While these results do not support a direct hierarchical regulation between gonadotropin-like signals and glycoprotein hormone orthologs on transcript level, they also do not exclude that such a regulation could exist at the level of peptide release.

Consequences of lysosomal α-mannosidase impairments in other species

One of the molecular consequences of knocking out the *Platynereis crz1/gnrhl1* gene that we reveal in this study is the strong reduction of lysosomal α-mannosidase. In humans, α-mannosidase deficiency leads to α-mannosidosis, a lysosomal storage disorder which causes the accumulation of mannose-rich polysaccharides, with consequent impairment of glycoprotein turnover and cellular functions (18). No defects in growth rate have been associated with patients affected by α-mannosidosis. Yet stunted growth characterizes guinea pigs with a deficiency in lysosomal α-mannosidase activity (19). Moreover, honeybee larvae show a developmental delay in pupal metamorphosis when fed with swainsonine (20), an alkaloid causing similar effects to α-mannosidosis (21). These findings are in line with the developmental delay we observe in *crz1-/-* worms, which may be caused by the accumulation of unprocessed mannose-rich glycans and related impairment in glycoprotein turnover (see ref. 18). Glycans processed by lysosomes can be used as additional energy sources, and in particular mannose can be converted into fructose-6-phosphate, which fuels both gluconeogenesis and glycolysis. Moreover, mannose-6-phosphate is also crucial as targeting signal, especially for proteins directed to lysosomes.

Corazonin- and GnRH signaling systems are involved in orchestrating life-history transitions

GnRH-like peptides have been already suggested to play a role in the regulation of developmental and life-history transitions in various animal systems. As mentioned in the introduction of our study, changes in GnRH signaling activity regulate puberty onset in vertebrates, an event which, on the one hand, requires the attainment of specific metabolic

conditions, and, on the other hand, involves major physiological and morphological changes. Moreover, in the tunicate *Ciona intestinalis*, GnRH-like hormones promote tail absorption in larvae, one of the two major events in ascidian metamorphosis (22). Finally, CRZ regulates density-dependent polyphenism in the locusts, *Schistocerca gregaria* and *Locusta migratoria* (23), whereas in the ant, *Harpegnatos saltator*, a CRZ peptide controls caste transition from worker to reproductive gamergate (a queen-like state) (24). It is interesting to note that CRZ promotes the release of preecdysis- and ecdysis-triggering hormones from the Inka cells of *Manduca sexta* (25). In line with this, a corazonin receptor was found to be expressed in the Y-organ (the gland which represents the source of steroid hormones and regulates moulting in decapod crustaceans) in the green shore crab, *Carcinus maenas* (26). Our observations in the *crz1/gnrhl1* mutant worms suggest that also outside arthropods, Corazonin signaling may engage in the regulation of key and particularly energy-demanding developmental transitions, potentially triggered also as a response to a physiologically-controlled metabolic stress (see (27–30)).

In vertebrates, GnRH has been proposed as a key endocrine regulator of both seasonal and semi-lunar/lunar reproduction (31–41). Yet, these notions are largely correlational, as it is hard to decouple the direct role that GnRH has in reproduction from its potential involvement as a downstream effector of seasonal and lunar timers. Indeed, outcomes of these infradian rhythms are often reproductive events requiring *per se* GnRH signaling (see ref. 39). The temporal separation between metamorphosis onset and spawning make the bristleworm an interesting model to distinguish between these processes. In fact, our expression data support the notion that GnRH-like signaling is more directly linked to lunar reproductive rhythmicity. In a broader perspective, our results thereby help to shed light on the still elusive endocrine basis of lunar-regulated life-histories.

Supplementary Methods

Worm culture and phenotypic analysis

For maturation timing experiments, data were retrieved from the analysis of culture boxes in which worms were growing at similar densities. To assess growth rate, we first measured the length of 2-months old worms kept in comparable density conditions and fed *ad libitum* (algae suspension). Then, 2-months old *crz1-/-* and +/+ worms were isolated in boxes with the same final density, and fed normally with the same amount of food (spinach and tetramin). After 3 months, worms were anesthetized and photographed. The length was measured using ImageJ software, and segments counted. For regeneration experiments, the 15 terminal segments of the tail were amputated from age-matched ~50 segments-long premature worms, and the number of re-grown segments counted every week under the microscope (after brief anesthesia) for 4 weeks. After the amputation, animals were placed in filtered sea water with the addition of ampicillin and streptinomycin (final concentration 62.5µg/mL and 250µg/mL, respectively) for two days. For the first (acute) feeding assay, we starved 12 age-matched premature worms of each genotype for 12 days, and then individually isolated them. We fed each worm with a single circular spinach disc, and we placed some leaves in control wells with no worms. At 1 and 3 days post leave administration, we used bright field microscopy, combined with image analysis, to quantify the area of residual leaves, and thereby estimated the consumed amount of spinach (see Fig. 3H). For the second one, after a milder starvation (5 days), animals were single isolated and fed continuously with new spinach circles as soon as the previous one was completely eaten. Worms were monitored for 5 weeks. In this case, data are shown as average number of days required to eat completely a circle leaf. Results were analyzed using Student's t-test: * $p<0.05$; ** $p<0.01$; *** $p<0.001$; **** $p<0.0001$.

qRT-PCR

Primers used were: *crz1/gnrhl1* F: 5'-TCCCAAATGACCTCATCGTC-3', R: 5'-AGTTGCAGACGGTCCATTTC-3'; *crz2/gnrhl2* F: 5'-GTGGTGTTATGGTGATTTTGTAGC-3', R: 5'-TCTCTCTTGTAAGGAAACAGACTTGA-3'; *gnrhl3* F: 5'-TCAACGTTTACCGTGCCATA-3', R: 5'- TCCTCCAATTTCTGCTGCAT-3'; *gnrh2/gnrhl4* F: 5'- GCATTATGTTCACAAGACGAAAA-3', *R*: 5'- TCGCACCACTTCTCTCTGAA-3'; *crzr* F: 5'-AGTGCCATCTTTAGTGCTCCA-3', R: 5'-AGCAAAGCAGAGATGATGGAA-3'; *gpa* F: 5'-TCACGAAGACACATAACGTGAA-3', R: 5'-GGCACAAGTCGAGCAACTG-3'; *gpb* F: 5'-CCTTGTTCAGCAATAGTTGAGGT-3', R: 5'-TGGAACCTTGTAATCAGGAATCT-3'; *cdc5* F: 5'-CCTATTGACATGGACGAAGAT G-3', R: 5'-TTCCCTGTGTGTTCGCAAG-3'; *lysosomal α-mannosidase* F: 5'-GCAACCTGTGACTGTGCAAT-3', R: 5'-CCCAGGATCAGTTCTTGGAC-3'; *tobi-like 1* F: 5'-TGGAGCCCATTACATTCCAT-3', R: 5'-ACAATTTGATTCGCATCTTCAG-3'; *tobi-like 2* F: 5'-TCCTGATCCTGCTCGAATG-3', R: 5'-GTGAATCCACGCACTCACTC-3'; *pepck* F: 5'-AATTCGAGAGAAGATGGAGTCG-3', R: 5'-CTAAGGGATCCGTTCTGCAC-3'; *glycogen synthase* F: 5'-TCGTCAGAGGACACTTCTATGG-3', R: 5'-CCAGCGATGAAGAAGTACAGC-3'; *sams* F: 5'-CAGCAACGGTGAAATAACCA-3', R: 5'-CATCACTCACTTGATCGCAAA-3'.

Tissue lysis and proteolytic digestion

Worm heads were resuspended in sample buffer (7.5 M urea, 1.5 M thiourea, 4 % CHAPS. 0.05 % SDS, 100 mM DTT) and sonicated. After centrifugation, protein concentrations were assessed by applying a Bradford assay (Bio-Rad-Laboratories, Vienna, Austria). Protein fractions were subjected to a filter-assisted proteolytic digestion with a modified version of the FASP protocol (42). In short, 20 µg protein were loaded onto a pre-wetted MWCO filter (Pall Austria Filter GmbH, Vienna, Austria) with a pore size of 3 kD, followed by reduction of disulfide bonds with dithiothreitol (DTT), alkylation with iodoacetamide (IAA) and washing steps with 50 mM ammonium bicarbonate buffer. Digestion of proteins was achieved by applying Trypsin/Lys-C with Mass Spec Grade quality (Promega, Mannheim, Germany) for

an overnight digest followed by the further addition of fresh enzyme and an additional incubation for four hours. Resulting peptides were eluted through the filter by centrifugation, and a clean-up step was performed using C-18 spin columns (Pierce, Thermo Fisher Scientific, Austria).

LC-MS/MS analysis

For LC-MS/MS analyses, samples were reconstituted in 5 µl 30 % formic acid (FA), supplemented with four synthetic peptide standards for internal quality control, and diluted with 40 µl mobile phase A (97.9 % H2O, 2 % ACN, 0.1 % FA). Of this solution, 5 µl were injected into a Dionex Ultimate 3000 nano LC-system coupled to a Q Exactive orbitrap mass spectrometer equipped with a nanospray ion source (Thermo Fisher Scientific, Austria). All samples were analyzed as technical replicates. As a pre-concentration step, peptides were loaded on a 2 cm x 75 µm C18 Pepmap100 pre-column (Thermo Fisher Scientific, Austria) at a flow rate of 10 µl/min using mobile phase A. Elution from the pre-column to a 50 cm x 75 µm Pepmap100 analytical column (Thermo Fisher Scientific, Austria) and subsequent separation was achieved at a flow rate of 300 nl/min using a gradient of 8 % to 40 % mobile phase B (79.9 % ACN, 2 % H2O, 0.1 % FA) over 235 min with a total chromatographic run time of 280 min. For mass spectrometric detection, MS scans were performed in the range from m/z 400-1400 at a resolution of 70000 (at m/z =200). MS/MS scans of the eight most abundant ions were achieved through HCD fragmentation at 30 % normalized collision energy and analyzed in the orbitrap at a resolution of 17500 (at m/z =200).

Proteome data analysis

For positive protein identification, as a minimum two peptides, at least one of them being unique, had to be detected. Trypsin/P was specified in the digestion mode. Peptide mass tolerance was set to 50 and 25 ppm for the first and the main search, respectively. The false discovery rate (FDR) was set to 0.01 both on peptide and protein level. Peptides were mapped against a reference proteome set established before (43). Carbamidomethylation

was set as fixed modification, methionine oxidation and N-terminal acetylation as variable modifications. Each peptide was allowed to have a maximum of two missed cleavages and two modifications. “Match between runs” was enabled and the alignment and match time window set to 10 and 1 min, respectively. The analysis of the quantitative protein abundance has been performed using the Perseus software platform (44), and the FDR (according to the Benjamini-Hochberg procedure) has been set at 0.2. Only proteins detected in at least four biological replicates have been considered in the analysis.

Phylogenetic studies and sequence analyses

Protein sequences of GnRH/AKH/CRZ preprohormones and receptors were retrieved from ref. 45 and the NCBI nr repository. Protein sequences of members of the Cys-Knot superfamily were retrieved from ref. 16 and NCBI nr repository. All other sequences were retrieved from the NCBI nr repository using similarity searches. Sequence alignments were performed using MUSCLE (46). Maximum likelihood trees were generated with IQ-TREE software (47), employing the ModelFinder option and a total of 1000 bootstraps; consensus trees were displayed and rooted using the Interactive Tree Of Life (ITOL) online tool (48).

Supplementary Figures

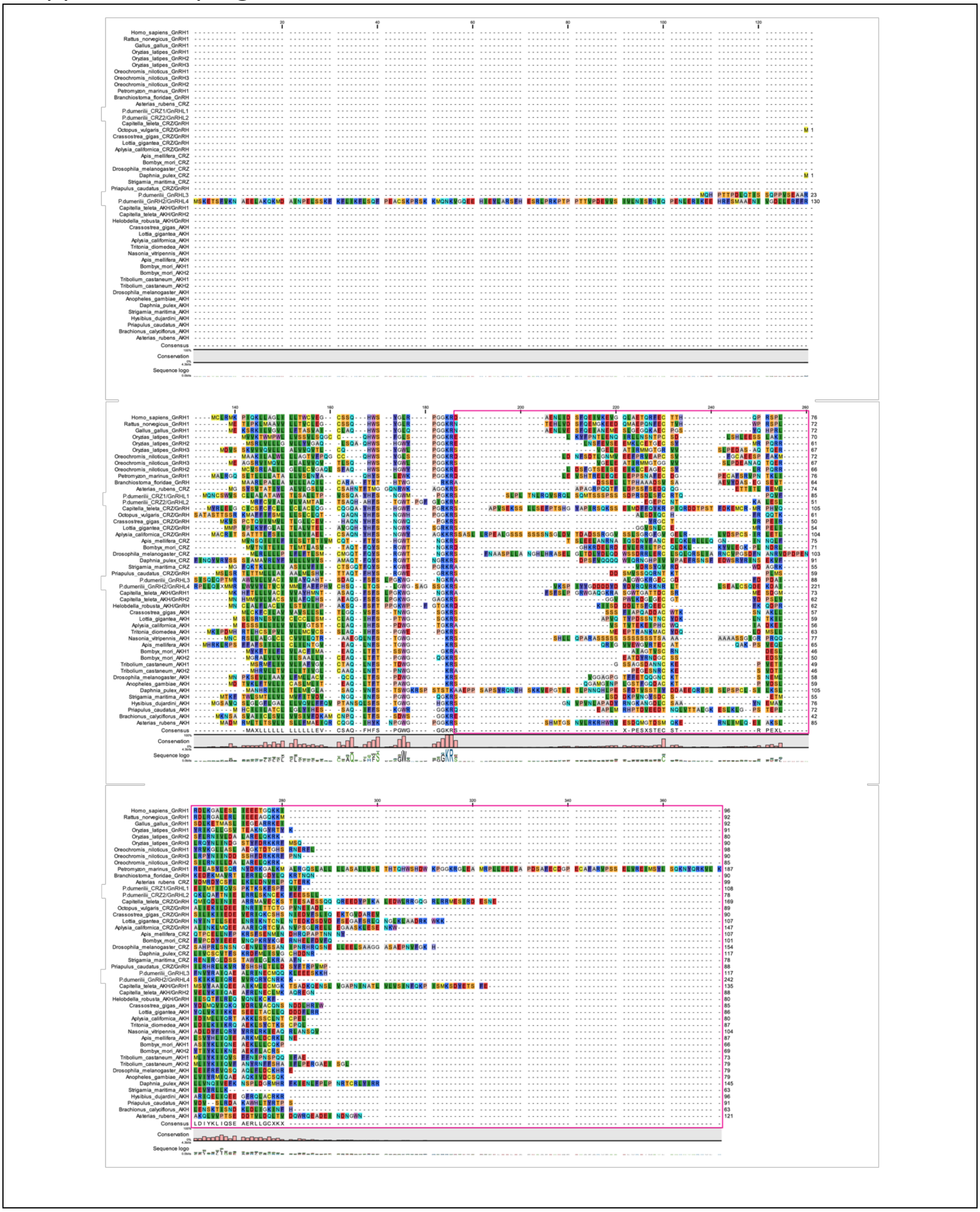

Fig. S1. Sequence alignment of GnRH/AKH/CRZ prepropeptides from different bilaterian taxa. The magenta box highlights the region corresponding to the GnRH-associated peptide (GAP), illustrating that there is no detectable conservation of GAP across the diverse groups.

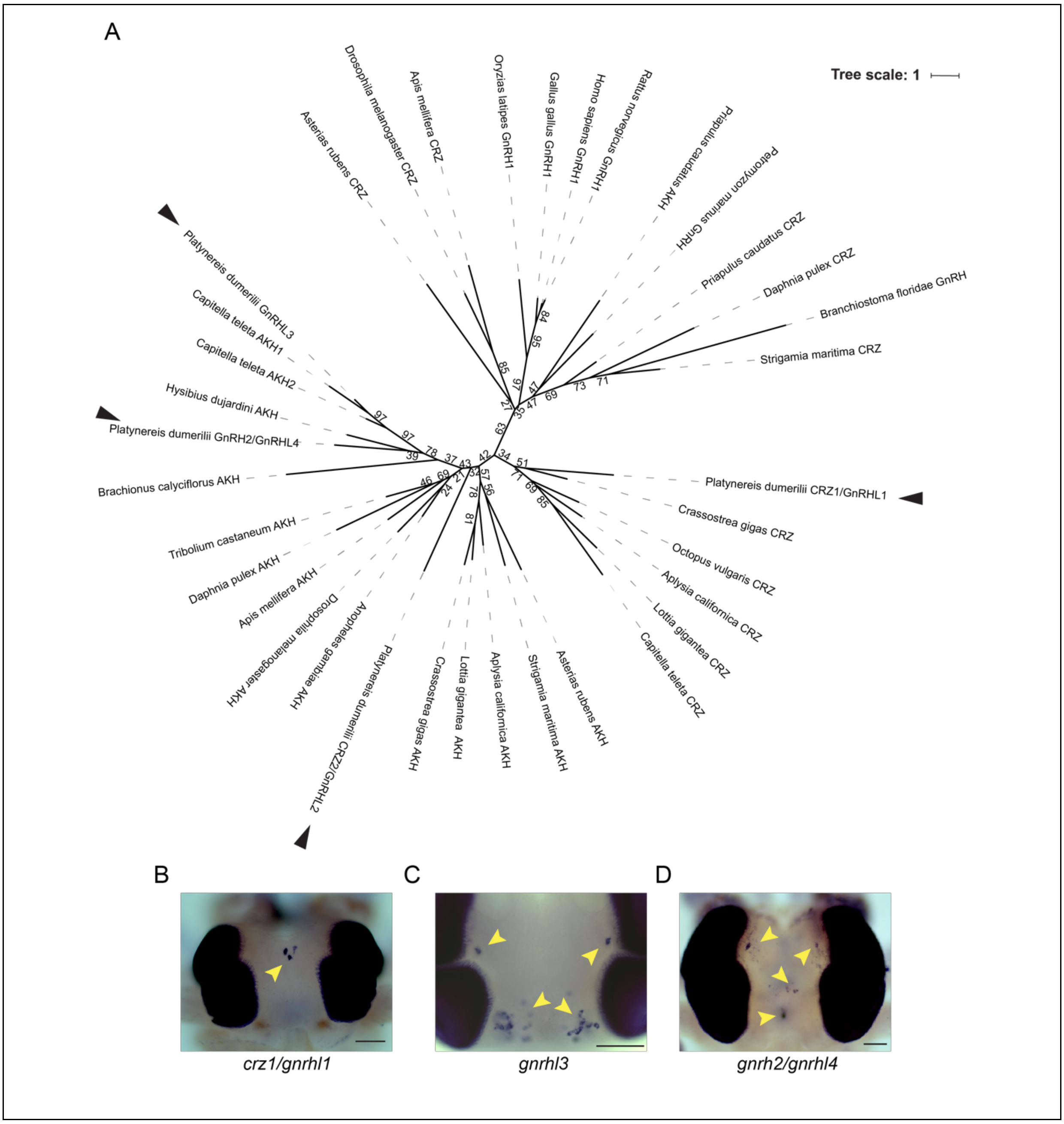

Fig. S2. Relationships and expression of *Platynereis* GnRH-like prepropeptides.

(A) Maximum Likelihood phylogeny of bilaterian GnRH-like preprohormones. Sequences were retrieved from (45) and the NCBI repository. **(B-D)** Whole-mount RNA *in situ* hybridization assays with riboprobes against *crz1/gnrhl1*, *gnrhl3*, and *gnrh2/gnrhl4* on head samples of sexually mature individuals. Yellow arrowheads demarcate distinct neuronal clusters of *gnrh-like* genes expressed in in the worm brain, suggesting at least partial functional diversification. Scale bar represents 100 µm.

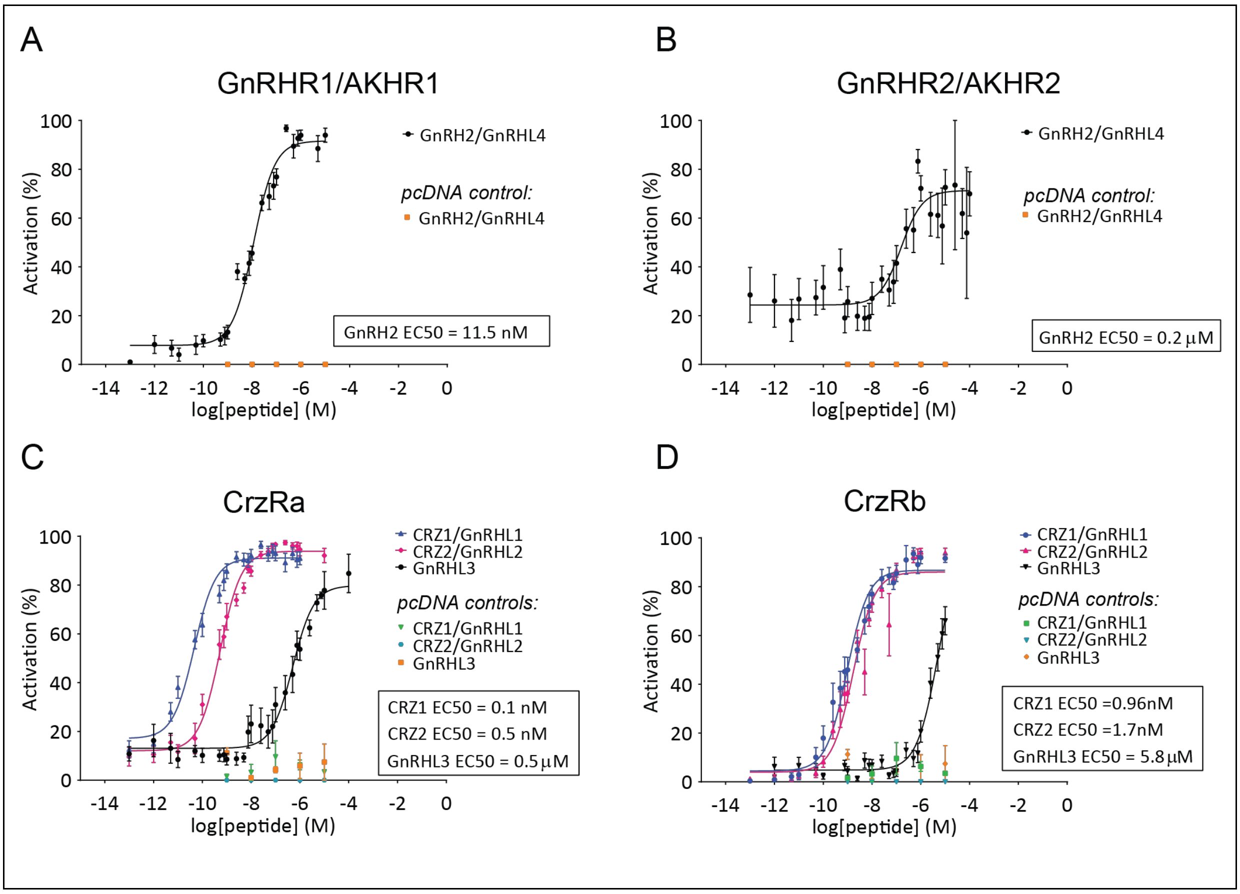

Fig. S3. Deorphanisation and ligand specificity of *Platynereis* GnRH-type receptors.

(A-D) Dose-response activation curves for the three *Platynereis* GnRH-like receptors characterized in this study, including two distinct CrzR isoforms. All receptors were tested with all of the ligands. The half maximal effective concentration (EC50 value) for each synthetic peptide is reported in the box for each graph. Negative controls (empty pcDNA3.1 vector) are shown for each ligand. **(A,B)** GnRHR1/AKHR1 and GnRHR2/AKHR2 are activated only by the addition of GnRH2/GnRHL4. **(C,D)** Both CrzR isoforms (a and b) are activated by CRZ1/GnRHL1 and CRZ2/GnRHL2 (see also ref. 4 for CrzRb). At sub-micromolar to micromolar range, CrzRa and CrzRb also respond to GnRHL3.

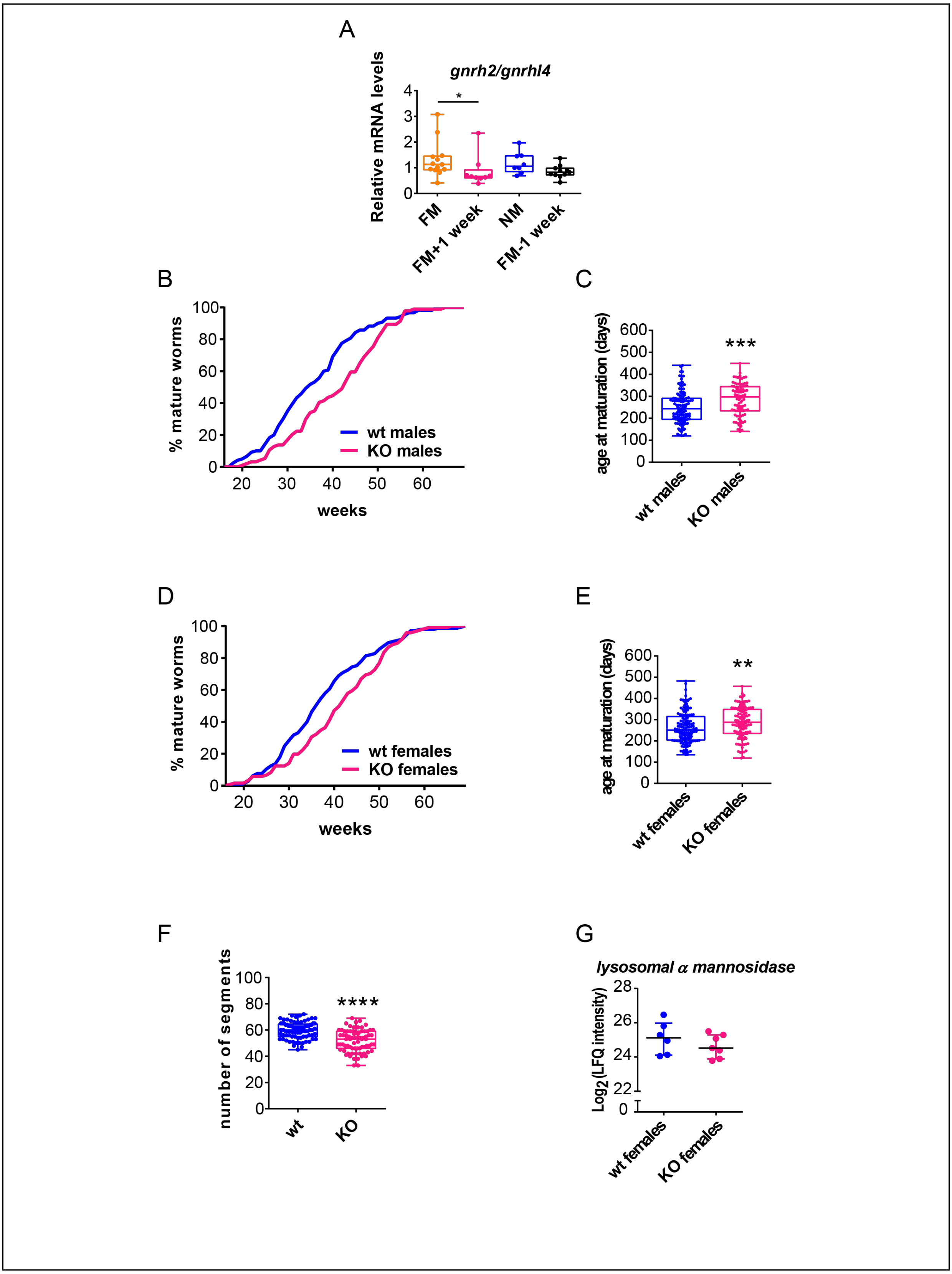

Fig. S4. Regulation of *gnrh2/gnrhl4*, and consequences of *crz1* knock-out on maturation and growth.

(A) Transcripts of *gnrh2/gnrhl4* are significantly upregulated in the head of late premature worms sampled during FM compared to animals collected in the week following FM. n=8-13 biological replicates. Statistical significance was tested using Kruskal-Wallis test followed by a Dunn's Multiple Comparison test to adjust the p-value for multiple testing. * $p<0.05$. 4-5 heads have been used for single biological replicate. Relative expression has been calculated using *cdc5* as reference gene, and data normalized to the expression of premature worms sampled during NM.

(B-E) In both *crz1* mutant (KO) males (B,C) and females (D,E) maturation is significantly delayed compared to respective wild-type (wt) controls. Data are presented as cumulative curves based on the percentage of mature animals (B and D), as well as plotting the age at which worms reached maturation (C and E). Wild-types are shown in blue, mutants (KO) in magenta. n≥92. Statistical significance was tested using t-test. **$p<0.01$; ***$p<0.001$.

(F) 4-months old premature *crz1-/-* mutants (KO) show a reduction in the number of segments compared to +/+ worms. n≥75 . Statistical significance was tested using t-test. ****$p<0.0001$.

(G) Extract from the analysis performed on *crz1-/-* (KO) and +/+ (wt) head proteome. The graph refers to the abundance of lysosomal α-mannosidase in the head proteome of *crz1+/+ and -/-* (KO) sexually mature females. n=6-7. Data are presented as LFQ intensity.

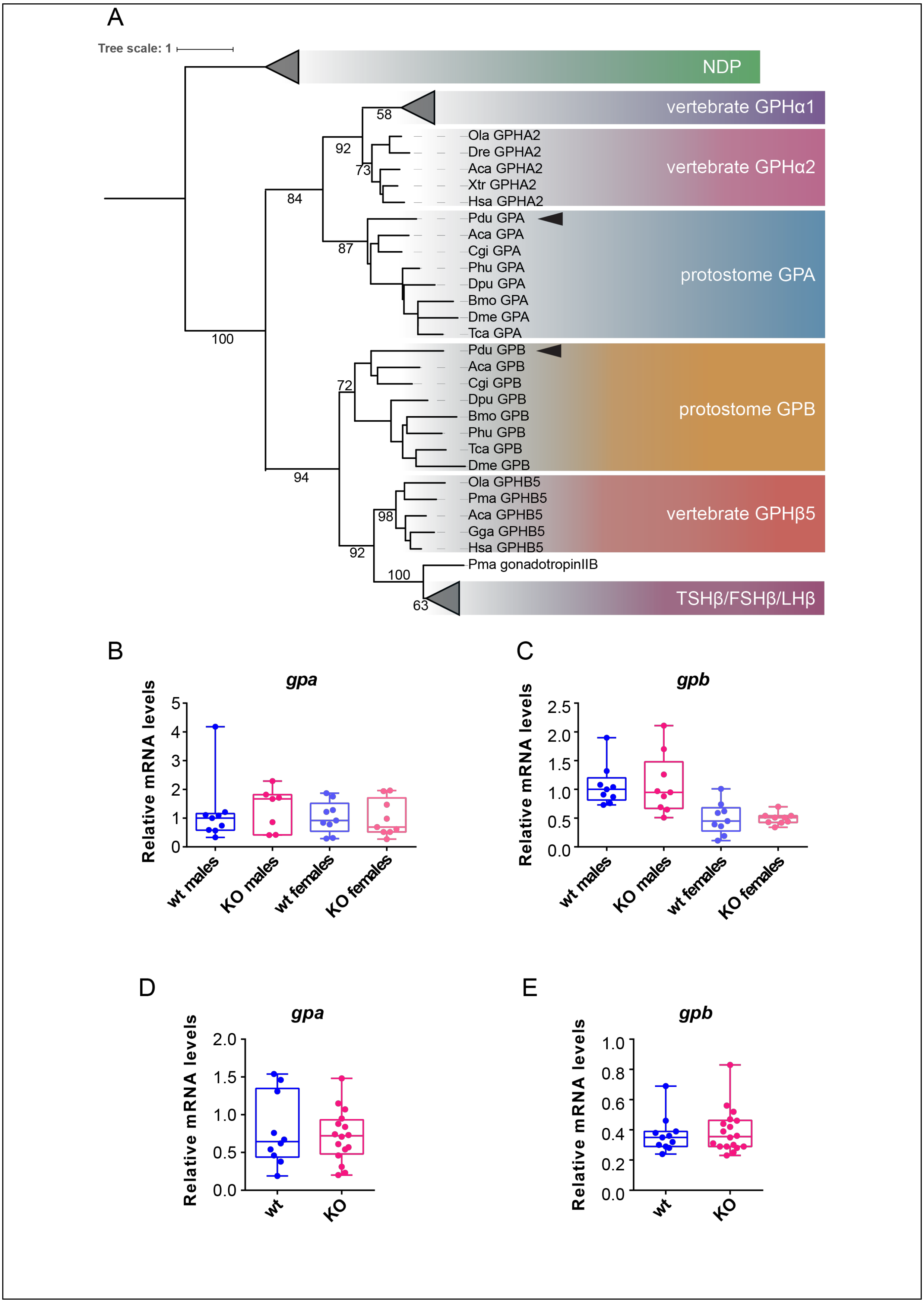

Fig. S5. Transcript levels of *Platynereis dumerilii* glycoprotein hormone α and β orthologs do not depend on functional *crz1/gnrhl1*.

(A) Maximum Likelihood phylogeny showing that the identified *Platynereis* glycoprotein hormone subunit homologs cluster together with other protostome glycoprotein α and β subunits. Sequences were retrieved from (16) and the NCBI repository. Bootstrap values, where not shown, are <28. Phylogeny rooted by the cysteine knot growth factor Norrin (NDP) branch. Color codes are used to distinguish relevant groups.

(B, C) Comparison of the relative expression levels of *Platynereis gpa* (B) and *gpb* (C) does not exhibit significant differences between *crz1* mutants (KO) and wild-type animals, in both mature males and females. **(D, E)** Corresponding analyses for the relative expression levels of *Platynereis gpa* (D) and *gpb* (E) in premature worms. 4-5 worm heads were used for each single biological replicate. Relative expression was calculated using *cdc5* as reference gene, and data normalized to the expression of *crz1 +/+* controls. n=10-13 biological replicates.

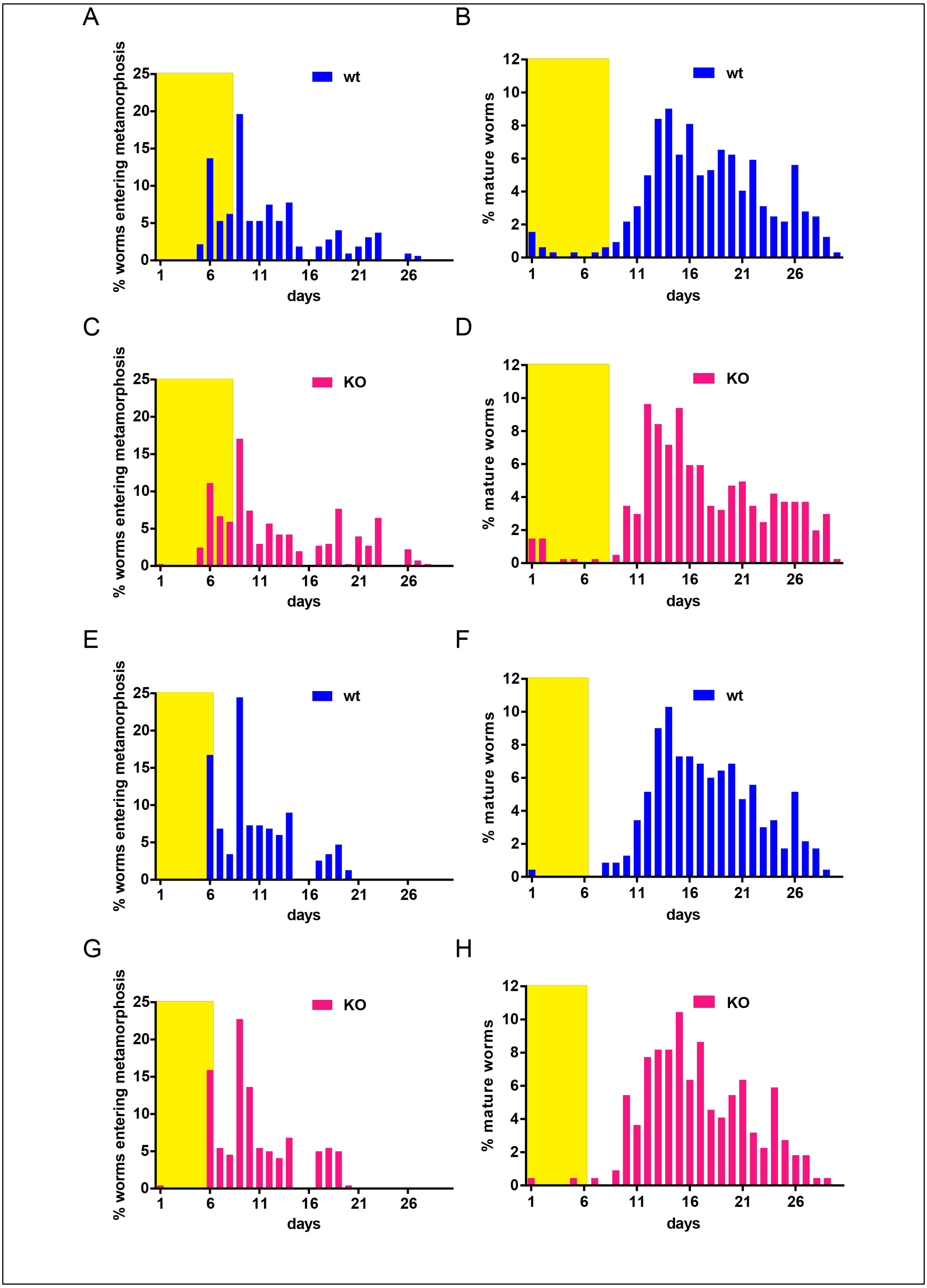

Fig. S6. ***crz1*** **knock-out strains retain a normal onset of metamorphosis and exhibit no major change in monthly spawning pattern.**

(A-H) Graphs on the left side of the panel (A, C, E, G) correspond to metamorphosis onset, whereas the ones on the right (B, D, F, H) refer to spawning. Blue graphs show data for wild-type (*crz1+/+*) animals, magenta graphs corresponding *crz1-/-* animals (KO). Data appear in two separate analyses (A-D, E-H) in order to faithfully represent the outcome of two large datasets differing in culture conditions: **(A-D)** Joint analysis of worms exposed to 6-8 nights of full moon (FM). **(E-H)** Analysis restricted to worms exposed to 6 nights of FM (the same condition experienced by the worms sampled for the qPCR analysis shown in Fig 2D-G). Data are presented as percentage of worms entering metamorphosis or spawning per day of the lunar calendar, where day 1 is set after the first night of full moon. Yellow rectangles represent the maximum extension of the period of full moon. Metamorphosis onset has been scored as the day where premature worms showed the first signs of color change (see Fig. 2A), unambiguously terminating their premature phase. $n \geq 214$.

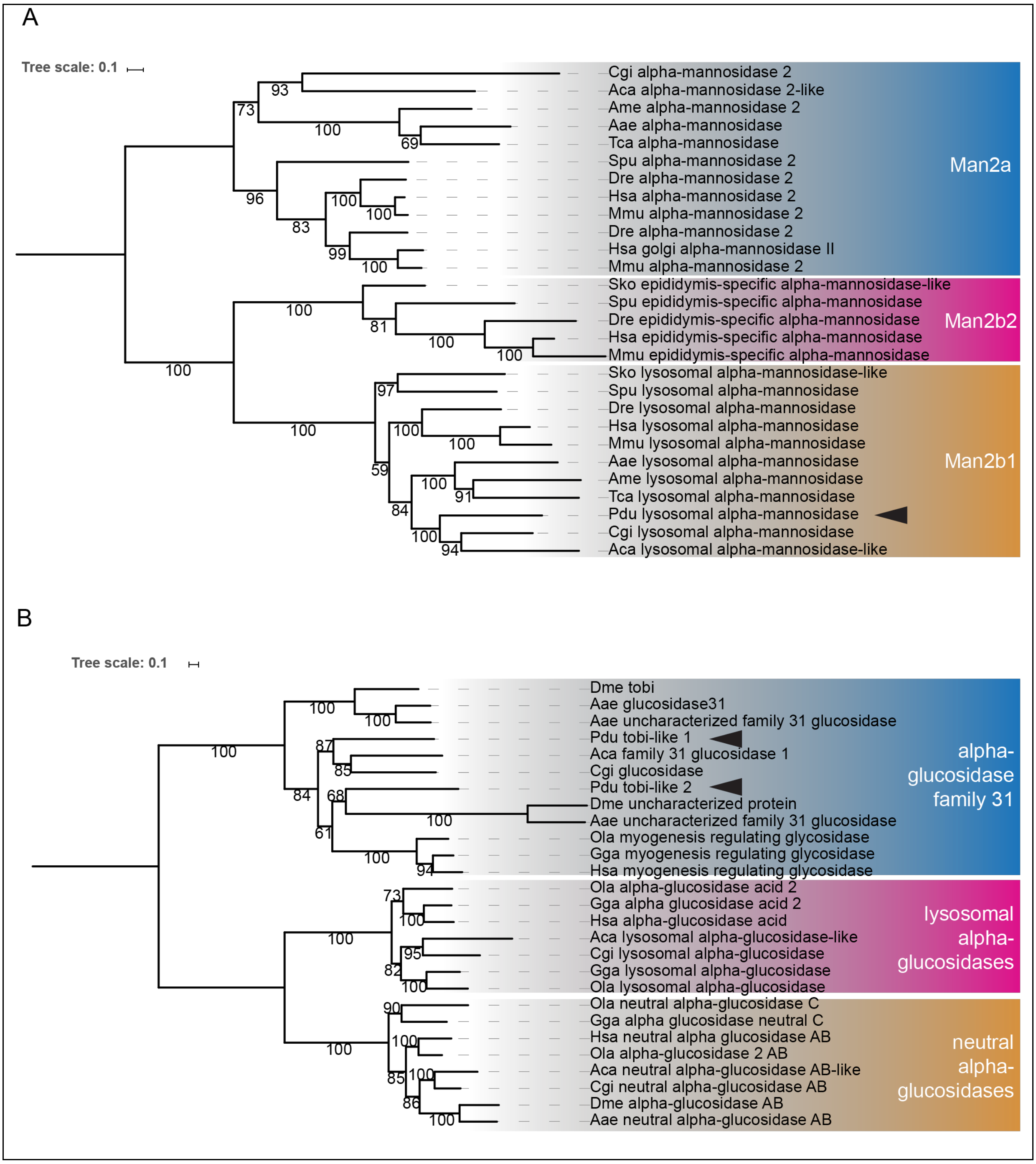

Fig. S7. Phylogenetic relationships of investigated metabolic enzymes.

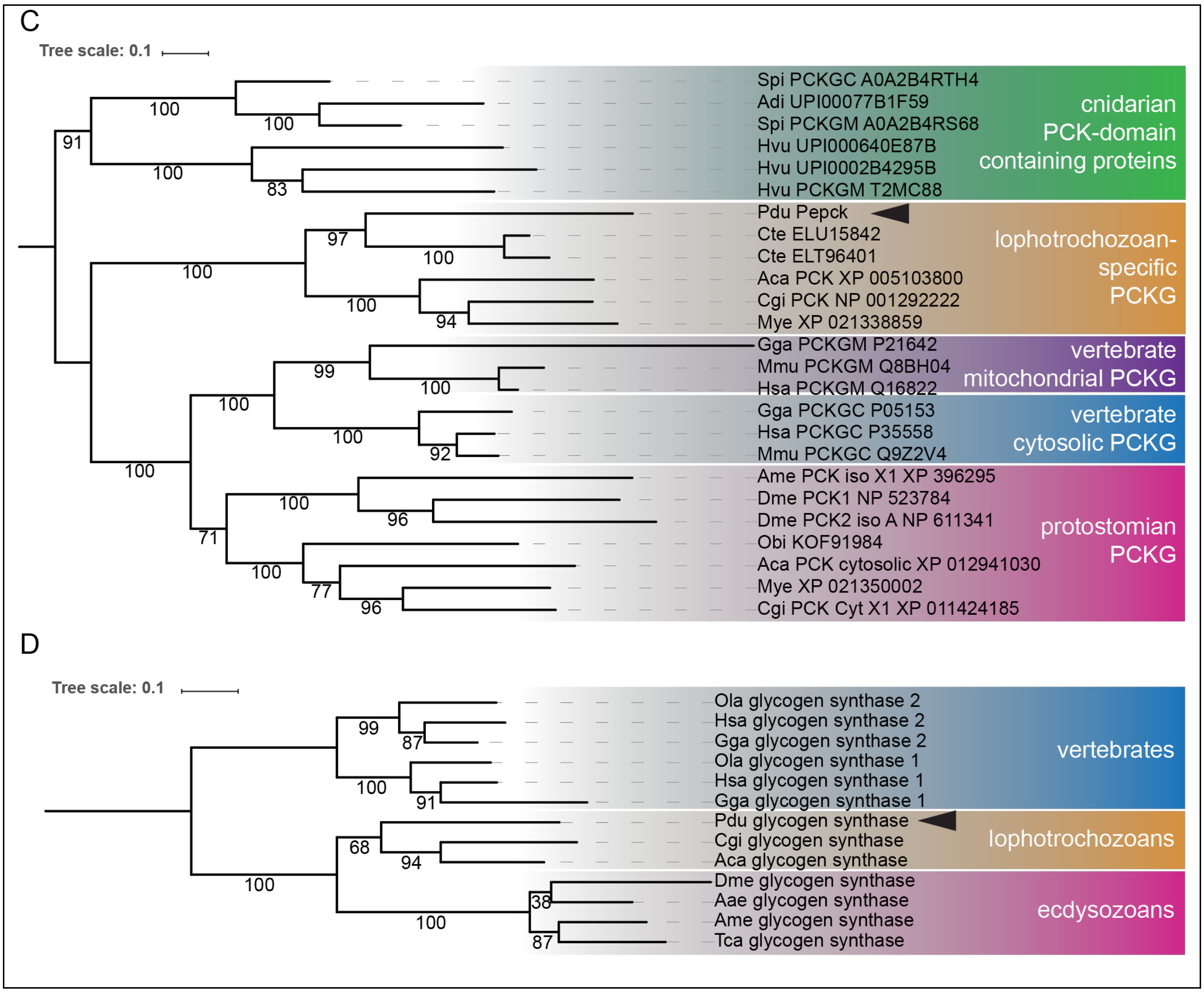

Fig. S7. Phylogenetic relationships of investigated metabolic enzymes (ct'd).

Maximum Likelihood phylogeny for metabolic enzymes investigated in this study; black arrowheads point at the respective *Platynereis* genes whose expression was analysed in this work (see Fig. 4). **(A)** Lysosomal alpha-mannosidases; **(B)** tobi-like/alpha-glucosidases; **(C)** phosphoenolpyruvate carboxykinases, and **(D)** glycogen synthases. Sequences were retrieved from the NCBI repository; color-codes were used to highlight specific subsets.

Tables S1-S3

Table S1. Overview of *Platynereis* GnRH-like ligands.

Name used in this study	Sequence	Alternative names	Precursor (gene ID)
CRZ1/GnRHL1	pQAYHFSNGWMPa	GnRH1[a], CRZ1[b]	MN537870[*]
CRZ2/GnRHL2	pQHAHFSTGWTPGFGIa	GnRH2[a], CRZ2[b]	MN537871[*]
GnRHL3	pQFSFSLPGKWGNa	AKH1[a], AKH[c]	KF515922.1
GnRH2/GnRHL4	pQLTQSLGWGSAGSSa	AKH2[a]	KF515936.1

Sources: [*]this study; [a] Conzelmann et al., 2013 (1); [b] Williams et al., 2017 (4); [c] Bauknecht et al., 2015 (5).

Table S2. Overview of *Platynereis* GnRH-like receptors

Name used in this study	Alternative names	Gene ID
Corazonin receptor isoform a (CrzRa)		MN537874[*]
Corazonin receptor isoform b (CrzRb)	GnRH receptor[a]	KY607913.1
Gonadotropin releasing hormone receptor 1/ Adipokinetic hormone receptor 1 (GnRHR1/AKHR1)		MN537872[*]
Gonadotropin releasing hormone receptor 2/ Adipokinetic hormone receptor 2 (GnRHR2/AKHR2)		MN537873[*]

Sources: [*]this study; [a] Williams et al., 2017 (4)

Table S3. Overview of additional *Platynereis* genes analysed in this study.

**Name used in this study**	**Gene ID**
Lysosomal α-mannosidase (laman/man2b1)	MN545477 *
Target of brain insulin-like 1 α-glucosidase (tobi-1)	MN545478 *
Target of brain insulin-like 2 α-glucosidase (tobi-2)	MN545479 *
Phosphoenolpyruvate carboxykinase-like (pepck)	MN545480 *
Glycogen synthase (gys)	MN545481 *
Glycoprotein hormone beta (gpb)	MN545476 *
Glycoprotein hormone alpha (gpa)	HAMO01057835.1

*this study

SI References

1. Conzelmann M, et al. (2013) The neuropeptide complement of the marine annelid *Platynereis dumerilii*. *BMC Genomics* 14(1):906.
2. Zandawala M, Tian S, Elphick MR (2018) The evolution and nomenclature of GnRH-type and corazonin-type neuropeptide signaling systems. *Gen Comp Endocrinol* 264:64–77.
3. Tsai PS (2018) Gonadotropin-releasing hormone by any other name would smell as sweet. *Gen Comp Endocrinol* 264:58–63.
4. Williams EA, et al. (2017) Synaptic and peptidergic connectome of a neurosecretory center in the annelid brain. *Elife* 6:1–22.
5. Bauknecht P, Jékely G (2015) Large-scale combinatorial deorphanization of *Platynereis* neuropeptide GPCRs. *Cell Rep* 12(4):684–693.
6. Schally A V, et al. (1971) Gonadotropin-releasing hormone: one polypeptide regulates secretion of luteinizing and follicle-stimulating hormones. *Science* 173(4001):1036–8.
7. McNeilly JR, Brown P, Clark AJ, McNeilly AS (1991) Gonadotrophin-releasing hormone modulation of gonadotrophins in the ewe: evidence for differential effects on gene expression and hormone secretion. *J Mol Endocrinol* 7(1):35–43.
8. McNeilly AS, Crawford JL, Taragnat C, Nicol L, McNeilly JR (2003) The differential secretion of FSH and LH: regulation through genes, feedback and packaging. *Reprod Suppl* 61:463–76.
9. Clayton RN (1993) Regulation of gonadotrophin subunit gene expression. *Hum Reprod* 8(suppl 2):29–36.
10. Dickey JT, Swanson P (2000) Effects of salmon gonadotropin-releasing hormone on follicle stimulating hormone secretion and subunit gene expression in coho salmon (*Oncorhynchus kisutch*). *Gen Comp Endocrinol* 118(3):436–449.
11. Kanda A, Takahashi T, Satake H, Minakata H (2006) Molecular and functional characterization of a novel gonadotropin-releasing-hormone receptor isolated from the

common octopus (*Octopus vulgaris*) . *Biochem J* 395(1):125–135.

12. Iwakoshi E, et al. (2002) Isolation and characterization of a GnRH-like peptide from *Octopus vulgaris*. *Biochem Biophys Res Commun* 291(5):1187–1193.
13. Bousfield, GR, Jia L, Ward DN (2006) Gonadotropins: chemistry and biosynthesis. *Knobil and Neill's Physiology of Reproduction* (3rd ed.) Vol.1, 1581-1634.
14. Bousfield GR, Dias JA (2011) Synthesis and secretion of gonadotropins including structure-function correlates. *Rev Endocr Metab Disord* 12(4):289–302.
15. Heyland A, et al. (2012) Distinct expression patterns of glycoprotein hormone subunits in the lophotrochozoan *Aplysia*: Implications for the evolution of neuroendocrine systems in animals. *Endocrinology* 153(11):5440–5451.
16. Roch GJ, Sherwood NM (2014) Glycoprotein hormones and their receptors emerged at the origin of metazoans. *Genome Biol Evol* 6(6):1466–1479.
17. Sower SA, et al. (2015) Emergence of an ancestral glycoprotein hormone in the pituitary of the sea lamprey, a basal vertebrate. *Endocrinology* 156(8):3026–3037.
18. Malm D, Nilssen Ø (2008) Alpha-mannosidosis. *Orphanet J Rare Dis* 3(1):21.
19. Crawley AC, Jones MZ, Bonning LE, Finnie JW, Hopwood JJ (1999) Alpha-mannosidosis in the guinea pig: a new animal model for lysosomal storage disorders. *Pediatr Res* 46(5):501–9.
20. Wedd L, Ashby R, Foret S, Maleszka R (2017) Developmental and loco-like effects of a swainsonine-induced inhibition of α -mannosidase in the honey bee, *Apis mellifera*. *PeerJ* 5:e3109.
21. Cenci di Bello I, Dorling P, Winchester B (1983) The storage products in genetic and swainsonine-induced human mannosidosis. *Biochem J* 215(3):693–6.
22. Kamiya C, et al. (2014) Nonreproductive role of gonadotropin-releasing hormone in the control of ascidian metamorphosis. *Dev Dyn* 243(12):1524–1535.
23. Sugahara R, Saeki S, Jouraku A, Shiotsuki T, Tanaka S (2015) Knockdown of the corazonin gene reveals its critical role in the control of gregarious characteristics in the desert locust. *J Insect Physiol* 79:80–87.

24. Gospocic J, et al. (2017) The neuropeptide corazonin controls social behavior and caste identity in ants. *Cell* 170(4):748-759.e12.

25. Kim Y-J, et al. (2004) Corazonin receptor signaling in ecdysis initiation. *Proc Natl Acad Sci* 101(17):6704–6709.

26. Alexander JL, et al. (2018) Functional characterization and signaling systems of corazonin and red pigment concentrating hormone in the green shore crab, *Carcinus maenas*. *Front Neurosci* 11(JAN):1–18.

27. Boerjan B, Verleyen P, Huybrechts J, Schoofs L, De Loof A (2010) In search for a common denominator for the diverse functions of arthropod corazonin: A role in the physiology of stress? *Gen Comp Endocrinol* 166(2):222–233.

28. Zhao Y, Bretz CA, Hawksworth SA, Hirsh J, Johnson EC (2010) Corazonin neurons function in sexually dimorphic circuitry that shape behavioral responses to stress in *Drosophila*. 5(2). doi:10.1371/journal.pone.0009141.

29. Kapan N, Lushchak O V, Luo J, Nässel DR (2012) Identified peptidergic neurons in the *Drosophila* brain regulate insulin-producing cells, stress responses and metabolism by coexpressed short neuropeptide F and corazonin. *Cell Mol Life Sci*. doi:10.1007/s00018-012-1097-z.

30. Kubrak OI, Lushchak O V., Zandawala M, Nässel DR (2016) Systemic corazonin signalling modulates stress responses and metabolism in *Drosophila*. *Open Biol* 6(11):160152.

31. Xiong J-J, Karsch FJ, Lehman MN (1997) Evidence for seasonal plasticity in the Gonadotropin-Releasing Hormone (GnRH) System of the ewe: changes in synaptic inputs onto GnRH neurons. *Endocrinology* 138(3):1240–1250.

32. Twan WH, Wu HF, Hwang JS, Lee YH, Chang CF (2005) Corals have already evolved the vertebrate-type hormone system in the sexual reproduction. *Fish Physiol Biochem* 31(2–3):111–115.

33. Ando H, Shahjahan M, Kitahashi T (2018) Periodic regulation of expression of genes for kisspeptin, gonadotropin-inhibitory hormone and their receptors in the grass puffer:

Implications in seasonal, daily and lunar rhythms of reproduction. *Gen Comp Endocrinol* 265(December 2017):149–153.

34. Smith JT, et al. (2008) Variation in kisspeptin and RFamide-related peptide (RFRP) expression and terminal connections to gonadotropin-releasing hormone neurons in the brain: a novel medium for seasonal breeding in the sheep. *Endocrinology* 149(11):5770–82.
35. Clarke IJ, Smith JT, Caraty A, Goodman RL, Lehman MN (2009) Kisspeptin and seasonality in sheep. *Peptides* 30(1):154–63.
36. Smith JT (2012) The role of kisspeptin and gonadotropin inhibitory hormone in the seasonal regulation of reproduction in sheep. *Domest Anim Endocrinol* 43(2):75–84.
37. Parhar I, Ogawa S, Kitahashi T (2012) RFamide peptides as mediators in environmental control of GnRH neurons. *Prog Neurobiol* 98(2):176–196.
38. Clarke I.J., Caraty A. (2013) Kisspeptin and Seasonality of Reproduction. In: Kauffman A., Smith J. (eds) Kisspeptin Signaling in Reproductive Biology. Advances in Experimental Medicine and Biology, vol 784. Springer, New York, NY.
39. Ando H, Shahjahan M, Hattori A (2013) Molecular neuroendocrine basis of lunar-related spawning in grass puffer. *Gen Comp Endocrinol* 181(1):211–214.
40. Ando H, et al. (2014) Diurnal and circadian oscillations in expression of kisspeptin, kisspeptin receptor and gonadotrophin-releasing hormone 2 genes in the grass puffer, a semilunar-synchronised spawner. *J Neuroendocrinol* 26(7):459–467.
41. Surbhi, Rastogi A, Rani S, Kumar V (2015) Seasonal plasticity in the peptide neuronal systems: Potential roles of gonadotrophin-releasing hormone, gonadotrophin-inhibiting hormone, neuropeptide Y and vasoactive intestinal peptide in the regulation of the reproductive axis in subtropical indian weaver birds. *J Neuroendocrinol* 27(5):357–369.
42. Wiśniewski JR, Zougman A, Nagaraj N, Mann M (2009) Universal sample preparation method for proteome analysis. *Nat Methods* 6(5):359–362.
43. Schenk S, et al. (2019) Combined transcriptome and proteome profiling reveals

specific molecular brain signatures for sex, maturation and circalunar clock phase. *Elife* 8. doi:10.7554/eLife.41556.

44. Tyanova S, et al. (2016) The Perseus computational platform for comprehensive analysis of (prote)omics data. *Nat Methods* 13(9):731–40.

45. Hauser F, Grimmelikhuijzen CJP (2014) Evolution of the AKH/corazonin/ACP/GnRH receptor superfamily and their ligands in the Protostomia. *Gen Comp Endocrinol* 209:35–49.

46. Edgar RC (2004) MUSCLE: a multiple sequence alignment method with reduced time and space complexity. *BMC Bioinformatics* 5(1):113.

47. Trifinopoulos J, Nguyen L-T, von Haeseler A, Minh BQ (2016) W-IQ-TREE: a fast online phylogenetic tool for maximum likelihood analysis. *Nucleic Acids Res* 44(W1):W232–W235.

48. Letunic I, Bork P (2016) Interactive tree of life (iTOL) v3: an online tool for the display and annotation of phylogenetic and other trees. *Nucleic Acids Res* 44(W1):W242-5.
